# History and progress of hypotheses and clinical trials for Alzheimer’s disease

**DOI:** 10.1038/s41392-019-0063-8

**Published:** 2019-08-23

**Authors:** Pei-Pei Liu, Yi Xie, Xiao-Yan Meng, Jian-Sheng Kang

**Affiliations:** Clinical Systems Biology Laboratories, The First Affiliated Hospital of Zhengzhou University, Zhengzhou, 450052 Henan China

**Keywords:** Neuroscience, Neurological disorders

## Abstract

Alzheimer’s disease (AD) is a neurodegenerative disease characterized by progressive memory loss along with neuropsychiatric symptoms and a decline in activities of daily life. Its main pathological features are cerebral atrophy, amyloid plaques, and neurofibrillary tangles in the brains of patients. There are various descriptive hypotheses regarding the causes of AD, including the cholinergic hypothesis, amyloid hypothesis, tau propagation hypothesis, mitochondrial cascade hypothesis, calcium homeostasis hypothesis, neurovascular hypothesis, inflammatory hypothesis, metal ion hypothesis, and lymphatic system hypothesis. However, the ultimate etiology of AD remains obscure. In this review, we discuss the main hypotheses of AD and related clinical trials. Wealthy puzzles and lessons have made it possible to develop explanatory theories and identify potential strategies for therapeutic interventions for AD. The combination of hypometabolism and autophagy deficiency is likely to be a causative factor for AD. We further propose that fluoxetine, a selective serotonin reuptake inhibitor, has the potential to treat AD.

## Introduction

Alzheimer’s disease (AD) is an irreversible progressive neurological disorder that is characterized by memory loss, the retardation of thinking and reasoning, and changes in personality and behaviors.^1,2^ AD seriously endangers the physical and mental health of the elderly. Aging is the biggest risk factor for the disease, the incidence of which doubles every 5 years after the age of 65.^3^ Approximately 40 million people over the age of 60 worldwide suffer from AD, and the number of patients is increasing, doubling every 20 years.^4–7^

In 1906, Alois Alzheimer presented his first signature case and the pathological features of the disease at the 37th convention of Southwestern German Psychiatrists. Later, in 1910, his coworker Emil Kraepelin named the disease in honor of his achievements. In the following years (from 1910 to 1963), researchers and physicians did not pay much attention to the disease until Robert Terry and Michael Kidd revived interest by performing electron microscopy of neuropathological lesions in 1963. Electron microscopy analysis showed that neurofibrillary tangles (NFTs) were present in brain biopsies from two patients with advanced AD.^8,9^ Since then, studies on the pathological features and mechanisms of AD and drug treatments for the disease have been conducted for more than half a century (from 1963 to present).^10^

Clinically, AD is divided into sporadic AD (SAD) and familial AD (FD). FD accounts for 1–5% of all AD cases.^11–15^ In the early 1990s, linkage analyses of early-onset FD determined that mutations in three genes, namely, amyloid-beta A4 precursor protein (*APP*), presenilin 1 (*PSEN1*), and presenilin 2 (*PSEN2*), are involved in FD. *PSEN1* mutations account for ~81% of FD cases, *APP* accounts for ~14%, and *PSEN2* accounts for ~6%.^11^ In addition to these three genes (*APP*, *PSEN1*, and *PSEN2*), more than 20 genetic risk loci for AD have been identified.^16,17^ The strongest genetic risk factor for AD is the *ε4* allele of apolipoprotein E (*APOE*).^18–21^ *APOE* is a class of proteins involved in lipid metabolism and is immunochemically colocalized to senile plaques, vascular amyloid deposits, and NFTs in AD. The *APOE* gene is located on chromosome 19q13.2 and is associated with late-onset FD. The *APOE* gene has three alleles, namely, *ε2*, *ε3*, and *ε4*, with frequencies of 8.4%, 77.9%, and 13.7%, respectively. The differences in APOE2 (Cys112, Cys158), APOE3 (Cys112, Arg158), and APOE4 (Arg112, Arg158) are limited to amino acid residues 112 and 158.^22–25^ Analyses of the frequencies of these *APOE* alleles among human populations have revealed that there is a significant association between APOE4 and late-onset FD (with an ε4 allele frequency of ~40% in AD), suggesting that ApoE4 may be an important susceptibility factor for the etiopathology of AD.^25–27^ Moreover, APOE4 can increase the neurotoxicity of β-amyloid (Aβ) and promote filament formation.^28^ The *APOE4* genotype influences the timing and amount of amyloid deposition in the human brain.^29^ Reelin signaling protects synapses against toxic Aβ through APOE receptors, which suggests that *APOE* is a potential target for AD therapy.^30^

The incidence of SAD accounts for more than 95% of all AD cases. Therefore, in this review, we focus our attention on recent SAD research and clinical trials. There are various descriptive hypotheses regarding the causes of SAD, including the cholinergic hypothesis,^31^ amyloid hypothesis,^32,33^ tau propagation hypothesis,^34^ mitochondrial cascade hypothesis,^35^ calcium homeostasis hypothesis,^36^ inflammatory hypothesis,^37^ neurovascular hypothesis,^38^ metal ion hypothesis,^39^ and lymphatic system hypothesis.^40^ In addition, there are many other factors that increase the risk for SAD, including family history,^41^ midlife hypertension,^42^ sleep disorders,^43^ midlife obesity,^44^ and oxidative stress.^45,46^ Interestingly, according to the latest evaluation of single-nucleotide polymorphisms (SNPs), Mukherjee et al. found 33 SNPs associated with AD and assigned people to six cognitively defined subgroups.^47^

At present, clinical drug treatments are mainly divided into two categories: acetylcholinesterase inhibitors (AChEIs), represented by donepezil, and the antagonist of N-methyl-D-aspartic acid (NMDA) receptor, represented by memantine (Table 1).^48^ As neurotransmitter regulators, these drugs can only relieve symptoms for a short time but cannot delay the progression of AD. Recent failures and the limited progress of therapeutics in phase III clinical trials suggest that it is time to consider alternative strategies for AD treatment.^49^

**Table 1:** Summary of pharmacological parameters of AD drugs

Drug	Time (approved by FDA)	Chemical class	Action	Type of inhibition	Route of administration	Indication	Status
Tacrine	1995	Alkaline	AChE inhibitor	Rapidly reversible	Oral or rectal	_	Withdrawal
Donepezil	1996	Piperidine	AChE inhibitor	Rapidly reversible	Oral	Mild- moderately (mod) AD	Approved
Rivastigmine	1997	Carbamate	AChE and BChE inhibitor	Pseudoreversible	Oral or transdermal patch	Mild-mod AD	Approved
Galantamine	2001	Phenanthrene alkaloid	AChE inhibitor	Rapidly reversible	Oral	Mild-mod AD	Approved
Memantine	2003	Glutamatergic modulator	NMDA antagonist	N/A	Oral	Mod-severe AD	Approved

**Fig. 1:**
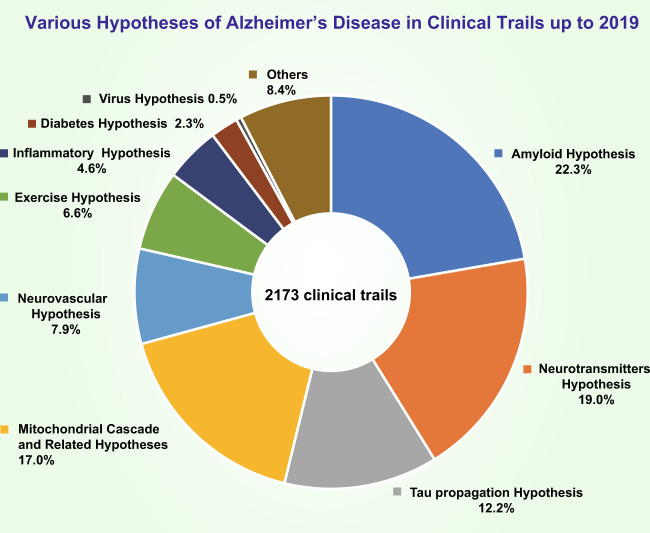
Percentage of clinical trials in which each of the various hypotheses for AD were tested up to 2019. The amyloid hypothesis was the most heavily tested (22.3% of trials); the neurotransmitter hypothesis was the second most tested (19.0% of trials); the percentage of trials that tested the tau propagation hypothesis was ~12.7%; 17.0% of trials tested the mitochondrial cascade hypothesis and related hypotheses; 7.9% of trials tested the neurovascular hypothesis; 6.6% of trials tested the exercise hypothesis; 4.6% of trials tested the inflammatory hypothesis; 0.5% of trials tested the virus hypothesis; and the other uncatalogued trials made up approximately 8.4% of all trials

In this review, we discuss the hypotheses of the molecular mechanisms of AD and related clinical trials (Fig. 1) and hope that these discussions will be helpful for developing explanatory theories and potential effective strategies for AD treatment.

## Hypotheses

### Cholinergic hypothesis

The cholinergic hypothesis was proposed by Peter Davies and A. J. F. Maloney in 1976^31^. They studied and compared the activities of the key enzymes involved in the synthesis of neurotransmitters, including acetylcholine, γ-aminobutyric acid, dopamine, noradrenaline, and 5-hydroxytryptamine, in 20 regions of AD and control brains. The activity of choline acetyltransferase in the AD brains was greatly reduced in the amygdala, hippocampus, and cortex, in which the concentration of acetylcholine was decreased at synapses.^50–52^ The activity of glutamic acid decarboxylase, tyrosine hydroxylase, aromatic amino acid decarboxylase, dopamine-β-hydroxylase, and monoamine oxidase in all the areas of the AD brains studied appeared to be well within the normal range. Choline acetyltransferase is a key enzyme in the synthesis of acetylcholine, and its catalytic activity requires these substrates: choline, acetyl-CoA, and adenosine triphosphate (ATP). This was the first time that the concept of AD was noted as a cholinergic system failure.^31,53^ This finding has also been reported in other neurological and psychiatric disorders, such as Parkinson’s disease (PD) and depression.^54,55^

AChEIs can alleviate cognitive impairment in AD patients by inhibiting the degradation of acetylcholine.^56–59^ Therefore, AChEIs have been used for more than 20 years since the FDA approved tacrine, the first drug for the treatment of AD, in 1995.^60^ Tacrine is a reversible AChEI. Because of its liver toxicity, the number of tacrine prescriptions dropped after other AChEIs were introduced, and the usage of tacrine has been largely discontinued. The second generations of AChEI drugs that are widely used at present include donepezil, rivastigmine, and galantamine.^61,62^ These drugs show fewer side effects and higher central selectivity and improve the cognition level of patients with mild to moderate AD. The daily living ability and overall function of patients treated with rivastigmine and galantamine are better than those treated with donepezil.^61–63^ According to the latest meta-analysis on the efficacy of AChEIs for treating the cognitive symptoms of dementia, AChEIs have modest effects on dementia in AD,^64^ but the effect is not continuous.^65,66^

In conclusion, the current clinical drugs used for the treatment of AD improve the quality of life of AD patients, but have no significant effect on the occurrence or progression of AD. In 2012, the French Pharmacoeconomic Committee assessed the medical benefit of these drugs and downgraded its rating of the medical benefit provided by AChEIs in AD from "major" to "low."^67^

### Amyloid hypothesis

The amyloid hypothesis was first proposed in 1991 by John Hardy and David Allsop.^32,33^ They found a pathogenic mutation in the Aβ precursor protein (APP) gene on chromosome 21, which suggested that APP mismetabolism and Aβ deposition were the primary events in AD. They thought that the pathological cascades in AD were Aβ deposition, tau phosphorylation, NFT formation, and neuronal death. The presence of Aβ deposits in an APP mutant (APP751) transgenic model supported the hypothesis and further contributed to shifting the amyloid hypothesis from a descriptive to a mechanistic hypothesis.^68,69^ Positron emission tomography (PET) imaging studies have suggested that ~30% of clinically normal older individuals have signs of Aβ accumulation.^70–73^

**Fig. 2:**
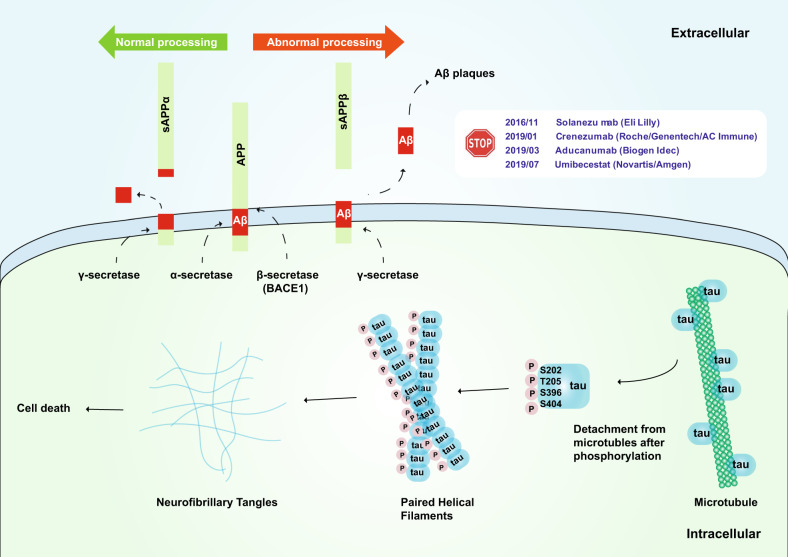
Schematic of the amyloid hypothesis and tau hypothesis. Upper: The transmembrane APP protein can be cleaved by two pathways. Under normal processing, APP is hydrolyzed by α-secretase and then by γ-secretase, which does not produce insoluble Aβ; under abnormal processing, APP is hydrolyzed by β secretase (BACE1) and then by γ secretase, which produces insoluble Aβ. Phase III clinical trials of solanezumab (Eli Lilly), crenezumab (Roche/Genentech/AC Immune), aducanumab (Biogen Idec), and umibecestat (Novartis/Amgen), which target the amyloid hypothesis, have all been terminated thus far. Lower: The tau protein can be hyperphosphorylated at amino residues Ser202, Thr205, Ser396, and Ser404 (which are responsible for tubulin binding), thereby leading to the release of tau from microtubules and the destabilization of microtubules. Hyperphosphorylated tau monomers aggregate to form complex oligomers and eventually neurofibrillary tangles, which may cause cell death

Aβ was first isolated by Glenner and Wong in 1984.^74^ Aβ may provide a strategy for diagnostic testing for AD and for understanding its pathogenesis.^74^ APP was first cloned and sequenced in 1987; APP consists of 695 amino acid residues and a glycosylated receptor located on the cell surface.^75,76^ Aβ is composed of 39–43 residues derived from multiple proteolytic cleavages of APP. APP is cleaved in two ways (Fig. 2). The first method is through the α pathway. APP is hydrolyzed by α-secretase and then by γ-secretase; this process does not produce insoluble Aβ. The second method is through the β pathway. APP is hydrolyzed by β-secretase (BACE1) and then by γ-secretase to produce insoluble Aβ. Under normal conditions, the Aβ protein is not produced since APP hydrolysis is mainly based on the α pathway. A small amount of APP is hydrolyzed via the second method, and the Aβ that produced is eliminated by the immune system. However, when some mutations, such as the Lys670Asn/Met671Leu (Swedish) and Ala673Val mutations near the BACE1 cleavage site, are present,^77,78^ APP is prone to hydrolysis by the β pathway, resulting in an excessive accumulation of insoluble Aβ and eventually the development of AD.^79,80^ However, the Ala673Thr mutation has been suggested to be protective.^81^

High concentrations of Aβ protein are neurotoxic to mature neurons because they cause dendritic and axonal atrophy followed by neuronal death.^82^ The levels of insoluble Aβ are correlated with the decline of cognition.^83^ In addition, Aβ inhibits hippocampal long-term potentiation (LTP) in vivo.^84^ Neurofibrillary degeneration is enhanced in tau and APP mutant transgenic mice.^85^ Transgenic mice that highly express human APP in the brain exhibit spontaneous seizures, which may be due to enhanced synaptic GABAergic inhibition and deficits in synaptic plasticity.^86^ Individuals with Aβ are prone to cognitive decline^87–89^ and symptomatic AD phenotypes.^90,91^

The current strategies for AD treatment based on the Aβ hypothesis are mainly divided into the following categories: β- and γ-secretase inhibitors, which are used to inhibit Aβ production; antiaggregation drugs (including metal chelators), which are used to inhibit Aβ aggregation; protease activity-regulating drugs, which are used to clear Aβ; and immunotherapy.^92^ We will discuss recent progress regarding immunotherapy and BACE1 inhibitors.

Aβ-targeting monoclonal antibodies (mAbs) are the major passive immunotherapy treatments for AD. For example, solanezumab (Eli Lilly), which can bind monomeric and soluble Aβ, failed to show curative effects in AD patients in phase III, although solanezumab effectively reduced free plasma Aβ concentrations by more than 90%.^93^ Gantenerumab (Roche/Genentech) is a mAb that binds oligomeric and fibrillar Aβ and can activate the microglia-mediated phagocytic clearance of plaques. However, it also failed in phase III.^94^ Crenezumab (Roche/Genentech/AC Immune) is a mAb that can bind to various Aβ, including monomers, oligomers, and fibrils. On January 30, 2019, Roche announced the termination of two phase III trials of crenezumab in AD patients. Aducanumab (Biogen Idec) is a mAb that targets aggregated forms of Aβ. Although aducanumab can significantly reduce Aβ deposition, Biogen and Eisai announced the discontinuation of trials of aducanumab on March 21, 2019. Together, the failure of these trials strongly suggests that it is better to treat Aβ deposits as a pathological feature rather than as part of a major mechanistic hypothesis.

BACE1 inhibitors aim to reduce Aβ and have been tested for years. However, no BACE1 inhibitors have passed clinical trials. Verubecestat (MK-8931, Merck & Co.) reduced Aβ levels by up to 90% in the cerebrospinal fluid (CSF) in AD. However, Merck no longer listed verubecestat in its research pipeline since verubecestat did not improve cognitive decline in AD patients and was associated with unfavorable side effects.^95^ Lanabecestat (AZD3293, AstraZeneca/Eli Lilly) is another BACE1 inhibitor that can lower CSF Aβ levels by up to 75%. However, on June 12, 2018, phase II/III trials of lanabecestat were discontinued due to a lack of efficacy. The BACE1 inhibitor atabecestat (JNJ-54861911, Janssen) induced a robust reduction in Aβ levels by up to 95% in a phase I trial. However, Janssen announced the discontinuation of this program on May 17, 2018. The latest news regarding the BACE inhibitor umibecestat (Novartis/Amgen) was released on July 11, 2019; it was announced that the evaluation of umibecestat was discontinued in phase II/III trials since an assessment demonstrated a worsening of cognitive function. Elenbecestat (E2609, Eisai) is another BACE1 inhibitor that can reduce CSF Aβ levels by up to 80%^96,97^ and is now in phase III trials (shown in Table 2). Although all BACE1 inhibitors seem to reduce CSF Aβ levels, the failure of trials of solanezumab, which can reduce free plasma Aβ concentrations by more than 90%,^93^ may be sufficient to lead us to pessimistic expectations, especially considering that the treatment worsened cognition and induced side effects.

**Table 2:** Current status of selected AD drugs in clinical trials

Drug	Developer	Mechanism of action	Stage	NCT number (https://clinicaltrials.gov)
Gantenerumab (RO4909832)	Roche/Genentech	Aβ-specific mAb	Phase III	NCT03443973
Solanezumab (LY2062430)	Eli Lilly	Aβ-specific mAb	Phase III	NCT02760602
Aducanumab (BIIB037)	Biogen Inc.	Aβ-specific mAb	Phase III (terminated)	NCT02484547
Crenezumab	Roche/AC Immune	Aβ-specific mAb	Phase III (terminated)	NCT02670083
AAB-003 (PF‑05236812)	Janssen/Pfizer	Aβ-specific mAb	Phase I (terminated)^457^	NCT01193608
Donanemab (N3pG‑Aβ)	Eli Lilly	Aβ-specific mAb	Phase II	NCT03367403
MEDI1814	AstraZeneca	Aβ-specific mAb	Phase I	NCT02036645
CAD106	Novartis	Aβ vaccine	Phase II	NCT01097096
ACI-24	AC Immune	Aβ vaccine	Phase I	NCT02738450
Verubecestat (MK-8931)	Merck & Co.	BACE1 inhibitor	Phase III (terminated)	NCT01953601
Lanabecestat (AZD3293)	AstraZeneca/Eli Lilly	BACE1 inhibitor	Phase III (terminated)	NCT02783573
Elenbecestat (E2609)	Eisai	BACE1 inhibitor	Phase III	NCT02036280
Atabecestat (JNJ-54861911)	Janssen	BACE1 inhibitor	Phase III (terminated)	NCT02569398
Umibecestat (CNP520)	Novartis/Amgen	BACE1 inhibitor	Phase II/III (terminated)	NCT03131453
LMTM (TRx0237)	TauRx Therapeutics	Tau-aggregation inhibitor	Phase III	NCT01626378
ACI-35	AC Immune/Janssen	Tau vaccine	Phase I	ISRCTN13033912
AADvac1	Axon Neuroscience SE	Tau vaccine	Phase II	NCT02579252
Naproxen	Seattle Institute for Biomedical and Clinical Research	Anti-inflammatory medication	Phase III (terminated)	NCT00007189
Celecoxib	Seattle Institute for Biomedical and Clinical Research	Anti-inflammatory medication	Phase III (terminated)	NCT00007189
Simvastatin	Charite University, Berlin, Germany	Cholesterol-lowering drug	Phase IV	NCT00842920
Valaciclovir	New York State Psychiatric Institute	Antiviral drug	Phase II	NCT03282916
Gemfibrozil	Gregory Jicha	MicroRNA-107 expression regulator	Early phase I	NCT02045056
Rosiglitazone	GlaxoSmithKline	Type II diabetes drug	Phase III (terminated)	NCT00490568
Neflamapimod (VX-745)	EIP Pharma, LLC	p38a kinase inhibitor	Phase II	NCT02423122
Ketasyn (AC-1202)	University of California, Los Angeles	Ketone body elevator	Phase IV	NCT01122329
GV-971	Shanghai Green Valley Pharmaceutical Co., Ltd.	Mannose oligosaccharide diacid	Phase III	NCT02293915

### Tau propagation hypothesis

Intracellular tau-containing NFTs are an important pathological feature of AD.^98,99^ NFTs are mainly formed by the aggregation of paired helical filaments (Fig. 2). Pathological NFTs are mainly composed of tau proteins, which are hyperphosphorylated.^100–103^ Tau proteins belong to a family of microtubule-binding proteins, and are heterogeneous in molecular weight. A main function of tau is to stabilize microtubules, which is particularly important for neurons since microtubules serve as highways for transporting cargo in dendrites and axons.^34,104^ Tau cDNA, which encodes a protein of 352 residues, was cloned and sequenced in 1988. RNA blot analysis has identified two major transcripts that are 6 and 2 kilobases long and are widely distributed in the brain.^105,106^ The alternative splicing of exons 2, 3, and 10 of the *tau* gene produces six tau isoforms in humans; the differential splicing of exon 10 leads to tau species that contain various microtubule-binding carboxyl terminals with repeats of three arginines (3R) or four arginines (4R).^107,108^ An equimolar ratio of 3R and 4R may be important for preventing tau from forming aggregates.^109^

The tau propagation hypothesis was introduced in 2009.^34^ The pathology of tau usually first appears in discrete and specific areas and later spreads to more regions of the brain. Aggregates of fibrillar and misfolded tau may propagate in a prion-like way through cells, eventually spreading through the brains of AD patients (Fig. 2). Clavaguera et al. demonstrated that tau can act as an endopathogen in vivo and in culture studies in vitro with a tau fragment.^104^ In their study, brain extracts isolated from P301S tau transgenic mice^110^ were injected into the brains (the hippocampus and cortical areas) of young ALZ17 mice, a tau transgenic mouse line that only develops late tau pathology.^111^ After the injection, the ALZ17 mice developed tau pathology quickly, whereas the brain extracts from wild-type mice or immunodepleted P301S mice, which were used as controls, had no effect. The causes of tau aggregation in sporadic tauopathies are not fully understood. Tau can be phosphorylated at multiple serine and threonine residues (Fig. 2).^112,113^ The gain- and loss-of-function of tau phosphorylation may be due to alterations in the activities of kinases or phosphatases that target tau, and thus, the toxicity of tau can be augmented as a result. Other posttranslational modifications can decrease tau phosphorylation or enhance the harmful states of tau. For example, serine–threonine modifications by O-glycosylation can reduce the extent of tau phosphorylation.^114,115^ Thus, tau hyperphosphorylation may partially result from a decrease in tau O-glycosylation. In addition, tau can also be phosphorylated at tyrosine residues,^116^ sumoylated and nitrated,^117^ but the exact roles of these tau modifications remain elusive.

According to the tau propagation hypothesis, abnormally phosphorylated tau proteins depolymerize microtubules and affect signal transmission within and between neurons.^101,103,118^ In addition, mutant forms of human tau cause enhanced neurotoxicity in *Drosophila melanogaster*.^119^ There may be cross-talk between the tau propagation hypothesis and the amyloid hypothesis. As mentioned earlier, among the risk loci for AD, *APOE* is the most robust factor for AD pathogenesis.^120^ Unlike other isoforms, APOE4 may increase Aβ by decreasing its clearance^121–123^ and enhancing tau hyperphosphorylation.^124–126^ GSK3 is one of the upstream factors that jointly regulates Aβ and tau. Increased GSK3 activity leads to the hyperphosphorylation of the tau protein.^126^ GSK3 overactivity may also affect the enzymatic processing of APP and thus increase the Aβ level.^127,128^ In addition, tau is essential for Aβ-induced neurotoxicity, and dendritic tau can mediate Aβ-induced synaptic toxicity and circuit abnormalities.^129^ Moreover, APP and tau act together to regulate iron homeostasis. APP can interact with ferroportin-1 to regulate the efflux of ferrous ions.^130,131^ As an intracellular microtubule-associated protein, tau can increase iron output by enhancing the transport of APP to the cell surface.^132^ Decreased APP trafficking to the cell surface accounts for iron accumulation in tau knockout neurons.^133,134^

As one of the most important hypotheses of AD, the tau propagation hypothesis has a wide range of impacts. Drugs that target the tau protein are divided into the following categories: tau assembly inhibitors, tau kinase inhibitors, O-GlcNAcase inhibitors, microtubule stabilizers, and immunotherapy drugs.^92^ Only a few agents have undergone proof-of-principle tests as tau kinase inhibitors, microtubule-stabilizing agents, and inhibitors of heat shock protein 90 (Hsp90), which stabilize GSK3β.^135,136^ In addition, some inhibitors of tau aggregation, such as TRx0237 (TauRx Therapeutics), are in clinical trials. The results of TRx 237–005 phase III clinical trials showed that the agent may be effective as a monotherapy since the brain atrophy rate of AD patients declined after 9 months of treatment.^137^ ACI-35 (AC Immune/Janssen) and AADvac1 (Axon Neuroscience SE) are vaccines that target the hyperphosphorylated tau protein, and the vaccines are still being evaluated in clinical trials^138^ (Table 2). Tau-directed therapies will inevitably face challenges similar to those presently encountered in Aβ-targeted trials. Overall, the effectiveness of tau-directed therapies remains to be tested in the future.

### Mitochondrial cascade hypothesis and related hypotheses (Fig. 3)

In 2004, Swerdlow and Khan first introduced the mitochondrial cascade hypothesis^35^ and stated that mitochondrial function may affect the expression and processing of APP and the accumulation of Aβ in SAD. The hypothesis includes three main parts. First, an individual's baseline mitochondrial function is defined by genetic inheritance. Second, the rate of age-associated mitochondrial changes is determined by inherited and environmental factors. Moreover, a decline in mitochondrial function or efficiency drives aging phenotypes.^139–141^ Third, the rate of change of mitochondrial function in individuals influences AD chronology.

**Fig. 3:**
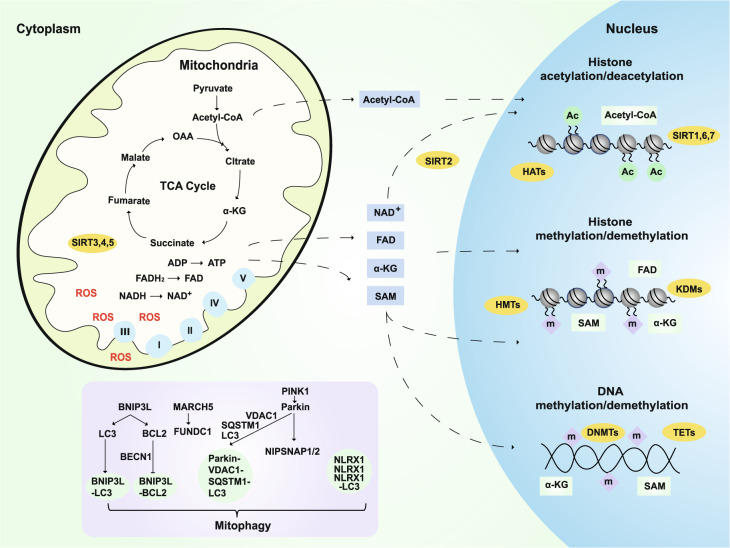
Mitochondrial cascade and related hypotheses. Mitochondria are the main contributors to ROS production, which is significantly increased in AD. The metabolites of mitochondrial TCA, such as pyruvate, fumarate, malate, OAA, and α-KG, not only directly regulate energy production but also play an important role in the epigenetic regulation of neurons and longevity.^164,173,187–189^ For example, SAM provides methyl groups for histone and DNA methyltransferases (HMTs and DNMTs).^165,166^ α-KG is a necessary cofactor for TET DNA methylases, histone demethylases (HDMs), and lysine demethylases KDMs/JMJDs.^167,168^ Mitochondria also regulate the levels and redox state of FAD, a cofactor of the histone demethylase LSD1.^175^ Dysfunctional mitochondria can be removed by mitophagy, which is also very important in the progression of AD. BNIP3L interacts with LC3 or GABARAP and regulates the recruitment of damaged mitochondria to phagophores. In addition, Beclin 1 is released from its interaction with Bcl-2 to activate autophagy after BNIP3L competes with it. PINK1 promotes autophagy by recruiting the E3 ligase PARK2. Then, VDAC1 is ubiquitinated and then binds to SQSTM1. SQSTM1 can interact with LC3 and target this complex to the autophagosome.^445^ *L. monocytogenes* can promote the aggregation of NLRX1 and the binding of LC3, thus activating mitophagy.^446^ The MARCH5-FUNDC1 axis mediates hypoxia-induced mitophagy.^447^ The mitochondrial proteins NIPSNAP1 and NIPSNAP2 can recruit autophagy receptors and bind to autophagy-related proteins.^448^ ROS: reactive oxygen species; TCA: tricarboxylic acid cycle; OAA: oxaloacetate; α-KG: α-ketoglutarate; SAM: S-adenosyl methionine; TET: ten–eleven translocation methylcytosine dioxygenase; FAD: flavin adenine dinucleotide

Oxidative stress is defined as “an imbalance in pro-oxidants and antioxidants with associated disruption of redox circuitry and macromolecular damage.”^142^ Oxidative stress is mainly caused by increased levels of reactive oxygen species (ROS) and/or reactive nitrogen species, including superoxide radical anions (O^2−^), hydrogen peroxide (H_2_O_2_), hydroxyl radicals (HO^−^), nitric oxide (NO), and peroxynitrite (ONOO^−^). In intact cells, ROS can be produced from multiple sources, including mitochondria, ER, peroxisomes, NADPH oxidases, and monoamine oxidases.^143,144^ In AD, neurons exhibit significantly increased oxidative damage and a reduced number of mitochondria,^145^ which are the main contributors to ROS generation among these ROS sources.^146,147^ The overproduction of ROS and/or an insufficient antioxidant defense can lead to oxidative stress.^148^ Before the onset of the clinical symptoms of AD and the appearance of Aβ pathology, there is evidence that the production of ROS increases due to mitochondrial damage.^148^ Both mtDNA and cytochrome oxidase levels increase in AD, and the number of intact mitochondria is significantly reduced in AD.^145^ Several key enzymes involved in oxidative metabolism, including dehydrogenase complexes for α-ketoglutarate (α-KG) and pyruvate, and cytochrome oxidase also show reduced expression or activity in AD.^149–154^ In addition, there is evidence in vitro and in vivo for a direct relationship between oxidative stress and neuronal dysfunction in AD.^155,156^ Aβ-dependent endocytosis is involved in reducing the number of NMDA receptors on the cell surface and synaptic plasticity in neurons and brain tissue in AD mice.^157^ Excessive Aβ may also trigger excitotoxicity and stress-related signaling pathways by increasing Ca^2+^ influx, increasing oxidative stress, and impairing energy metabolism.^158^

Although the majority of efforts have been focused on genetic variations and their roles in disease etiology, it has been postulated that epigenetic dysfunction may also be involved in AD.^159,160^ Indeed, there is growing evidence that epigenetic dysregulation is linked to AD.^161–163^ Mitochondrial metabolites are required for epigenetic modifications, such as the methylation of DNA and the methylation and acetylation of histones.^164^ AD brains exhibited a global reduction in DNA modifications, including 5-methylcytosine and 5-hydroxymethylcytosine.^165–168^ S-adenosyl methionine (SAM) provides a methyl group for histones and DNA methyltransferases in the nucleus. SAM is generated and maintained by coupling one-carbon metabolism and mitochondrial energy metabolism.^169,170^ α-KG, which is generated by the tricarboxylic acid cycle (TCA) cycle in mitochondria and the cytosol, is a cofactor of ten–eleven translocation methylcytosine dioxygenase DNA methylases, histone demethylases (HDMs) and the lysine demethylases KDMs/JMJDs.^171,172^ However, the activities of KDMs/JMJDs and TETs can be inhibited by fumarate, succinate, and 2-hydroxyglutarate.^173^ Mutations that affect the succinate dehydrogenase complex and fumarate hydratase can induce the accumulation of succinate and fumarate, respectively.^174^ Oxidized flavin adenine dinucleotide (FAD) is an essential cofactor of the HDM LSD1, a member of the KDM family.^175^ In addition, acetyl-CoA, the source of acetyl groups that are consumed by histone acetyltransferases, is generated by ATP citrate lyase and pyruvate dehydrogenase in the cytosol and mitochondria, respectively.^176^ In addition, oxidized nicotinamide adenine dinucleotide (NAD^+^) is a cofactor for sirtuins (SIRTs), a family of deacetylases that includes nuclear-localized SIRT1, SIRT6, and SIRT7, cytosolic SIRT2, and three mitochondrial SIRTs (SIRT3, SIRT4, and SIRT5) (Fig. 3). Therefore, the activities of SIRTs are sensitive and are regulated by cellular NAD^+^ pools.^177^ As summarized by Fang, NAD^+^ replenishment can enhance autophagy/mitophagy mainly through SIRT1 or SIRT3; meanwhile, SIRT6 and SIRT7 induce autophagy through the inhibition of mTOR; NAD^+^ may also inhibit autophagy/mitophagy through SIRT2, SIRT4, SIRT5, and poly(ADP-ribose) polymerases.^178^ In short, mitochondrial dysfunction can partially explain the epigenetic dysregulation in aging and AD.

Dysfunctional mitochondria can be removed by mitophagy, a term that was first coined by Dr Lemasters in 2005.^179^ Since then, mitophagy has been linked to various diseases, including neurodegenerative disorders such as PD^180^ and Huntington's disease (HD),^181^ as well as normal physiological aging.^182^ Mitophagosomes can effectively degrade their internalized cargo by fusing with lysosomes during axonal retrotransport.^183^ Fang et al. demonstrated that neuronal mitophagy is impaired in AD.^184^ Mitophagy stimulation can reverse memory impairment, diminish insoluble Aβ 1–42 and Aβ 1–40 through the microglial phagocytosis of extracellular Aβ plaques, and abolish AD-related tau hyperphosphorylation.^184^ Therefore, deficiencies in mitophagy may have a pivotal role in AD etiology and may be a potential therapeutic target.^178,184–186^

The metabolites of mitochondrial TCA, such as pyruvate, fumarate, malate, oxaloacetate (OAA), and α-KG, have been demonstrated to extend lifespan when fed to *C. elegans*.^173,187–189^ Wilkins et al. found that OAA enhances the energy metabolism of neuronal cells.^190^ Moreover, OAA can also activate mitochondrial biogenesis in the brain, reduce inflammation, and stimulate neurogenesis.^191^ The application of OAA in AD was also investigated by Swerdlow et al., and the results showed that 100-mg OAA capsules did not result in an elevation of OAA in the blood^192^; higher doses up to 2 g per day were also evaluated in clinical studies, but no results have been posted or published yet.

Clinical trials related to the mitochondrial cascade hypothesis and related hypotheses account for 17.0% of all clinical trials (Fig. 1). Based on the above, the mitochondrial cascade hypothesis and related hypotheses (Fig. 3) may link other hypotheses, including the cholinergic hypothesis, amyloid hypothesis, and tau propagation hypothesis.

### Calcium homeostasis and NMDA hypotheses

The calcium homeostasis hypothesis was proposed in 1992 by Mattson et al. They found that Aβ can elevate intracellular calcium levels and render neurons more vulnerable to environmental stimuli.^36^ The involvement of calcium in AD was first suggested long ago by Khachaturian,^193^ and since then, there are many efforts to clarify this hypothesis.^194–196^ Calcineurin can trigger reactive/inflammatory processes in astrocytes, which are upregulated in AD models.^197^ In addition, calcium homeostasis is closely related to learning and memory. Rapid autopsies of the postmortem human brain have suggested that calcineurin/nuclear factor of activated T-cells signaling is selectively altered in AD and is involved in driving Aβ-mediated cognitive decline.^198^ The evidence indicates that calcium homeostasis may be associated with the development of AD.^193,199^

Memantine, a noncompetitive antagonist of NMDA glutamate receptors in the brain was approved for marketing in Europe in 2002 and received US FDA approval in 2003.^200,201^ Memantine is not an AChEI. The functional mechanism of memantine likely involves blocking current flow (especial calcium currents) through NMDA receptors and reducing the excitotoxic effects of glutamate.^202^ Memantine is also an antagonist of type 3 serotonergic (5-HT_3_) receptors and nicotinic acetylcholine receptors, but it does not bind other receptors, such as adrenergic, dopamine, and GABA receptors. The inhibition of NMDA receptors can also reduce the inhibition of α-secretase and thus inhibit the production of Aβ.^203^ However, the French Pharmacoeconomic Committee downgraded its rating of the medical benefit provided by memantine in AD from "major" to "low,"^67^ which was also supported by a recent meta-analysis.^64^

### Neurovascular hypothesis

The homeostasis of the microenvironment and metabolism in the brain relies on substrate delivery and the drainage of waste through the blood; neurons, astrocytes, and vascular cells form a delicate functional unit that supports the integrity of brain structure and function.^204–206^ Vascular dysregulation leads to brain dysfunction and disease. Alterations in cerebrovascular function are features of both cerebrovascular pathologies and neurodegenerative diseases, including AD.^38^ In 1994, it was demonstrated that the cerebral microvasculature is damaged in AD.^207^ Aβ can induce the constriction of the cerebral arteries.^208^ In an AD mouse model, neocortical microcirculation is impaired before Aβ accumulation.^209,210^ Neuroimaging studies in AD patients have demonstrated that neurovascular dysfunction is found before the onset of neurodegeneration.^211–214^ In addition to aberrant angiogenesis and the senescence of the cerebrovascular system, the faulty clearance of Aβ across the blood–brain barrier (BBB) can initiate neurovascular uncoupling and vessel regression and consequently cause brain hypoperfusion, brain hypoxia, and neurovascular inflammation. Eventually, BBB compromise and a chemical imbalance in the neuronal environment lead to neuronal dysfunction and loss.^215^ In mice that overexpress APP, impairment in the neocortical microcirculation is observed. The cerebrovascular effects of Aβ in dementia may involve alterations in cerebral blood flow and neuronal dysfunction.^209^ Moreover, neurovascular dysfunction may also play a role in the etiology of AD.

Many factors can lead to changes in the neurovasculature, which in turn affect the occurrence and progression of AD. Of these factors, hyperlipidemia is one of the most important. During the last two decades, growing evidence has shown that a high cholesterol level may increase the risk of AD. In one test, higher levels of low-density lipoprotein (LDL) or total cholesterol were correlated with lower scores on the MMSE (modified mini mental state exam) in nondemented patients. High total cholesterol levels in midlife increase the risk of AD nearly threefold: the odds ratio (OR) is 2.8 (95% confidence interval, CI: 1.2–6.7).^216^ Midlife obesity is also a risk factor for AD,^217^ and midlife adiposity may predict an earlier onset of dementia and Aβ accumulation.^218^ Adipose tissue secretes some inflammation factors, such as tumor necrosis factor (TNF-α), interleukin-1 (IL-1), and interleukin-6, in obesity,^219^ and these factors may induce insulin resistance, produce Aβ deposits, and stimulate excessive tau phosphorylation.^220^

A hyperglycemic state is another risk factor. Type 2 diabetic patients (T2D) have an increased risk of dementia,^221^ both vascular dementia (VD) and AD. In the largest and latest meta-analysis of T2D and dementia risk, data from 6184 individuals with diabetes and 38,350 without diabetes were pooled and analyzed.^222^ The relative risk (RR) for dementia was 1.51 (95%CI: 1.31–1.74). The results of the analyses further suggested that there are two common subtypes of dementia: AD and VD. The results suggested that T2D conferred an RR of 2.48 (95%CI: 2.08–2.96) for VD and 1.46 (95%CI: 1.20–1.77) for AD.^222^ Insulin resistance is a common feature of T2D and SAD. Accumulating evidence supports the involvement of impaired insulin signaling in AD progression. Insulin levels and insulin receptor expression are reduced in AD brains.^223^ However, plasma insulin and Aβ levels are both increased in AD patients, suggesting that a decrease in insulin clearance may increase plasma Aβ levels. Blocking insulin signaling in the brain through the intracerebroventricular administration of STZ (the diabetogenic drug streptozotocin) resulted in various pathological features that resemble those found in human SAD, while the administration of insulin and glucose enhances learning and memory in AD patients.^224,225^

Many institutions have conducted clinical trials of statins, drugs that are used to lower blood cholesterol, for the treatment of AD. However, in a phase IV clinical trial, simvastatin failed to reduce Aβ-42 and tau levels in the CSF. The results suggested that the use of statins for the treatment of AD requires more evidence.^226^ To test the hyperglycemic hypothesis, rosiglitazone (RSG), a drug used for the treatment for type II diabetes mellitus, was evaluated. RSG XR had no effect in a phase III trial.^227^ In addition, hypertension has also been linked to worse cognition and hypometabolism in AD. AD patients with hypertension exhibit worse cognitive function (on the AD assessment scale-cognitive subscale, *P* = 0.038) and a higher burden of neuropsychiatric symptoms (on the neuropsychiatric inventory questionnaire, *P* = 0.016) than those without hypertension.^228^ As an antihypertensive medication, ramipril is a specific angiotensin-converting enzyme inhibitor; however, ramipril was tested and failed in a pilot clinical trial.^229^

Therefore, trial failures of treatments related to the neurovascular hypothesis and related hypotheses suggest that these hypotheses alone may not be sufficient to explain the etiology of AD.

### Inflammatory hypothesis

The inflammatory responses of microglia and astrocytes in the central nervous system (CNS) also play important roles in the development of AD.^230–232^ Microglial cells are brain-specific macrophages in the CNS, and they make up 10–15% all brain cells.^233^ Microglia cells exhibit higher activity in AD patients than in the control group.^234^ The concentration of aggregated microglial cells near senile plaques and neurons with NFTs in AD patients is usually 2–5 times higher than that in normal individuals. Inflammatory factors that are expressed by microglia and histocompatibility complexes also cause inflammation.^235^ In vitro studies have linked Aβ pathology in AD to neuroinflammation. It has been shown that Aβ possesses a synergistic effect on the cytokine-induced activation of microglia.^236^ Two studies have confirmed that Aβ can induce glial activation in vivo.^237,238^ The fibrillar conformation of Aβ seems to be crucial for such activation.^239^ In AD patients, Aβ can bind to microglia cells through the CD36-TLR4-TLR6 receptor complex and the NLRP3 inflammatory complex, destroy cells, release inflammation-inducing factors, such as TNF-α, and cause immune responses. In addition to increased levels of TNF-α, increased levels of the inflammatory cytokines IL-1β, TGF-β, IL-12, and IL-18 in the CNS are also correlated with AD progression and increase damage in the brains of AD patients.^240^ Interestingly, CD22 is a B-cell receptor that functions as a negative regulator of phagocytosis. The functional decline of aged microglia may result from the upregulation of CD22; thus, the inhibition of CD22 can enhance the clearance of debris and fibrils, including Aβ oligomers, in vivo, and this process may be potentially beneficial for the treatment of AD.^241^

Considerable evidence suggests that the use of anti-inflammatory drugs may be linked with a reduced occurrence of AD. The ability of naproxen and celecoxib to delay or prevent the onset of AD and cognitive decline was evaluated in phase III clinical trials. However, therapeutic efficacy analysis indicated that naproxen and celecoxib do not exert a greater benefit compared with that of placebo. In addition, the naproxen and celecoxib groups experienced more adverse events, including hypertension, gastrointestinal, and vascular or cardiac problems, so these phase III clinical trials were discontinued.^242^ A clinical trial of lornoxicam in AD patients was also terminated due to a lack of efficacy. These failures suggest that the clinical application of anti-inflammatory drugs for AD treatment needs to be further validated (Table 2).

### Metal ion hypothesis

Metal ions that play functional roles in organisms are classified as biometals, while other metal ions are inert or toxic.^243,244^ The dyshomeostasis of any metal ion in the body usually leads to disease. In the CNS, biometals, such as copper, zinc, and iron, are required to act as cofactors for enzymatic activity, mitochondrial function, and neuronal function.^245,246^ In healthy brains, free metal ions are stringently regulated and kept at a very low level.^247^

Biometal ions are involved in Aβ aggregation and toxicity. In the first study that evaluated biometals and Aβ, which was published by Bush et al. in 1994, zinc was linked to Aβ. The potential link between biometals and AD has been intensively studied.^39,248–250^ There is evidence of the dyshomeostasis of biometals in AD brains. Biometals, especially zinc and copper, are directly coordinated by Aβ, and biometals such as iron can reach a high concentration (~1 mM) in plaques.^251,252^ In the serum, the levels of copper, which are not associated with ceruloplasmin, are elevated in AD patients. Moreover, a higher copper content in the serum is associated with lower MMSE scores.^253,254^ In the serum of AD patients, the levels of Zn^2+^ ions are decreased compared with those in age-matched controls, whereas the concentration of Zn^2+^ is elevated in the CSF.^255^

The important role of biometals in Aβ formation has been reported in various animal models. For example, the role of Cu^2+^ in Aβ formation was demonstrated in a cholesterol-fed rabbit model of AD.^256^ Administering trace amounts of Cu^2+^ in drinking water was sufficient to induce Aβ accumulation, the consequent formation of plaques, and deficits in learning.^256^ On the other hand, Cu^2+^ also plays a beneficial role. For example, transgenic mice that overexpress mutant human APP and are treated with Cu^2+^ show a reduction in Aβ and do not exhibit a lethal phenotype.^257^ In contrast, in *Drosophila* that specifically express human Aβ in the eye, dietary zinc and copper increase Aβ-associated damage, while different chelators of biometals demonstrate favorable effects.^258^

During normal aging, the gradual accumulation of iron is observed in some brain areas, such as the substantia nigra, putamen, globus pallidus, and caudate nucleus.^259–263^ An increase in the level of iron in AD brains was first demonstrated in 1953.^264^ More recently, through the use of magnetic resonance imaging (MRI), iron accumulation was found in AD and was shown to be mainly localized to certain brain areas, such as the parietal cortex, motor cortex, and hippocampus.^265–272^ Studies of gene mutations that affect the metabolism of iron have suggested that the dyshomeostasis of iron plays a role in neuronal death, such as the neuronal death that occurs in neurodegenerative disorders such as AD.^273–277^ Iron overload accelerates neuronal Aβ production and consequently worsens cognitive decline in a transgenic AD mice.^278^ There is evidence that the levels of labile iron can directly affect APP production via iron regulatory element.^279^ As a potent source of highly toxic hydroxyl radicals, redox-active iron is actively associated with senile plaques and NFTs.^280^

As the most common nutrient deficiency in the world, iron deficiency is also frequently observed and reported in AD.^281^ Iron is present in polynuclear iron–sulfur (Fe/S) centers and hemoproteins. Mitochondrial complexes I–III require Fe/S clusters, and complexes II–IV need hemoproteins for electron transfer and the oxidative phosphorylation of the respiratory chain.^282^ Thus, iron deficiency may partially account for hypometabolism in AD since women with iron deficiency anemia have a higher prevalence of dementia.^283^ Interestingly, iron deficiency and iron accumulation in AD seem paradoxical. One potential explanation is that tau differentially regulates the motor proteins dynein and kinesin; specifically, tau may preferentially inhibit kinesin, which transports cargo toward the cell periphery.^284^ Tau is distributed in a proximal-to-distal gradient with a low concentration in the cell body.^284–287^ When tau is hyperphosphorylated, it is released from the distal microtubules into the neuronal axon and soma, and thus inhibits kinesin activity and prevents the transport of iron-containing cargo and other cargo (including mitochondria) to the neuronal periphery; this may result in the accumulation of mtDNA and iron accumulations in the soma of neurons in AD^145,280^ and deficiencies in mitochondria and iron homeostasis in the white matter of the brain. Iron-targeted therapies were recently updated and reviewed.^288^ Similar to the amyloid hypothesis, the conjecture that the therapeutic chelation of iron ions is an effective approach for treating AD remains widespread despite a lack of evidence of any clinical benefits.^288^

Aluminum (Al), the most abundant metal in the earth’s crust, is a nonessential metal ion in organisms. The role of Al in AD needs to be further elucidated. Exley et al. hypothesized that Al is associated with Aβ in AD brains and Al can precipitate Aβ in vitro into fibrillar structures; in addition, Al is known to increase the Aβ burden in the brains of treated animals, which may be due to a direct or indirect effect on Aβ anabolism and catabolism.^289,290^

Biometals may play various roles in AD and may influence the pathogenesis directly or indirectly. For example, biometals indirectly influence energy metabolism and APP processing,^249^ while cellular iron levels can directly regulate *APP* through IREs identified in the 5′ -UTR of mRNA.^291,292^

### Lymphatic system hypothesis

The lymphatic network and the blood vasculature are essential for fluid balance in the body.^293,294^ Below the human skull, the meninges, a three-layer membrane that envelopes the brain, contains a network of lymphatic vessels. This meningeal lymphatic system was first discovered in 1787, and interest in this system has been revived recently.^295–297^ Proteins, metabolites, and waste produced by the brain flow through the interstitial fluid (ISF) and reach the CSF, which circulates through the ventricles and brain meninges.^298^ In the classical form of transvascular removal, metabolic waste and other molecules in these fluids are drained from the brain, are transported across capillary walls, and cross the BBB.^298,299^ Thrane et al.’s found that, in addition to transvascular removal, perivascular removal, in which the blood vasculature allows the CSF to flow into or exit the brain along the para-arterial space or via paravenous routes, occurs and that aquaporin-4 water channels that are expressed in astrocytes are essential for CSF–ISF exchange along the perivascular pathway.^300,301^ This perivascular route is called the glymphatic system.^302,303^

During aging, impairments in the transvascular/perivascular removal of waste may result in Aβ accumulation in the brain.^40,304^ Animals that lack aquaporin-4 channels show a 70% decrease in the ability to remove large solutes, such as Aβ.^305,306^ Da Masquita et al.’s investigated the importance of meningeal lymphatics for Aβ production in AD mouse models. They found that ablating meningeal lymphatics leads to Aβ accumulation in the meninges, accelerates Aβ deposition, and induces cognitive deficits. These findings are consistent with Aβ accumulation observed in the meninges of AD patients. Strategies for promoting the growth of meningeal lymphatic vessels may have the potential to enhance the clearance of Aβ and lessen the deposition of Aβ,^307,308^ but this remains to be further validated.

### Other hypotheses

In addition to the above hypotheses, there are many other factors that can affect the occurrence of AD. For a long time (at least 60 years), investigators have suspected that microbes may be involved in the onset and progression of AD, this was hypothesized by Sjogren et al. beginning in 1952.^309^ In addition to McLachlan et al.’s proposal in 1980,^310^ several investigators have proposed that AD may be caused by a viral form of herpes simplex.^311–314^ There have been intensive reports suggesting that AD may be associated with various bacterial and viral pathogens,^315–317^ especially herpesviridae (including HSV-1,^318,319^ EBV, HCMV, HHV-6A, and HHV-7^314,320^). However, these studies did not determine the underlying mechanisms or identify a robust association with a specific viral species. Recent reports have suggested that Aβ aggregation and deposition may be stimulated by different classes of microbes as a part of the innate immune response. Microbes trigger amyloidosis, and newly generated Aβ acts as an antimicrobial peptide to coat microbial particles to fight the infection.^321–323^ Valaciclovir, an antiviral drug that is used for the management of herpes simplex and herpes zoster, is now in a phase II trial for AD (Table 2).

MicroRNAs (miRNAs) are involved in posttranscriptional gene regulation.^324–327^ The decreased expression of miRNA-107 (miR-107) in AD may accelerate disease progression by regulating the expression of BACE1.^328^ In SAD patients, the expression of miR-29a/b-1 is inversely correlated with BACE1 expression.^329^ Only one clinical trial related to miRNAs is underway. Gregory Jicha launched a phase I trial to assess the safety and efficacy of gemfibrozil in modulating miR-107 levels for the prevention of AD in subjects with cognitive impairment (Table 2).

Mannose oligosaccharide diacid (GV-971) was developed by researchers at the Shanghai Institute of Medicine, the Chinese Academy of Sciences, the Ocean University of China, and the Shanghai Green Valley Pharmaceutical Co., Ltd. GV-971 is an oceanic oligosaccharide molecule extracted from seaweed. GV-971 may capture multiple fragments of Aβ in multiple sites and multiple states, inhibit the formation of Aβ filaments, and depolymerize filaments into nontoxic monomers^330,331^; however, an understanding of the exact mechanism is still lacking. GV-971 has been reported to improve learning and memory in Aβ-treated mice.^332^ In phase II trials, GV-971 improved cognition in AD patients.^333^ In addition, a phase III clinical trial of GV-971 finished with positive results, and it is on its way to the market in China (Table 2).

Interestingly, a pilot clinical trial that included 120 nondemented elderly Chinese individuals (ages 60–79) living in Shanghai compared the effects of interventions (such as walking, Tai Chi, and social interaction) on cognition and whole brain volume, as determined by a neuropsychological battery and MRI scans.^334^ The results showed that Tai Chi and social interaction were beneficial, but walking had no effect. Therefore, in addition to promising drugs, a healthy lifestyle can delay the progression of AD.

### Opinions

The whole brain atrophy rate is −0.67 to −0.8% per year in adulthood.^335^ Freeman et al.’s results demonstrated that, although the frontal and temporal regions of the cortex undergoing thinning, the total number of neurons remains relatively constant from age 56 to age 103. However, there is a reduction in the number of hippocampal neurons in AD but not in normal aging. The loss of neuronal structural complexity may contribute to the thinning that occurs with aging.^336^ The integrity of neurons and dendritic structures is the basis for maintaining the normal function of neurons.^337–339^ Brain atrophy affects the function of neurons, which in turn impairs signal transmission and causes movement disorders, cognitive disorders etc.^340–343^ Brain atrophy has been shown to be a key pathological change in AD.^344–347^ In particular, the annual atrophy rate of the hippocampus in AD patients (−3.98 ± 1.92%) is two to four times that of the atrophy rate in healthy individuals (−1.55 ± 1.38%). At the same time, the annual increase in the temporal lobe volume of the lateral ventricle in AD patients (14.16 ± 8.47%) is significantly greater than that in healthy individuals (6.15 ± 7.69%).^348^ The ratio of the volume of the lateral ventricle to the volume of the hippocampus may be a reliable measurement for evaluating AD since the ratio can minimize variances and fluctuations in clinical data and may be a more objective and sensitive method for diagnosis and evaluating AD. In 1975, brain atrophy and a reduction in perfusion were detected in AD patients.^349^ In 1980, atrophy of hippocampal neurons and abnormal brain metabolism were first discovered in AD patients with PET.^350^ Brain volume reduction in patients with AD is significantly associated with dementia severity and cognitive disturbances as well as neuropsychiatric symptoms.^351^ The development of broad-spectrum drugs that target brain atrophy, a common feature of neurodegenerative diseases, is still ongoing. In our previous work, RAS–RAF–MEK signaling was demonstrated to protect hippocampal neurons from atrophy caused by dynein dysfunction and mitochondrial hypometabolism (tetramethylrhodamine ethyl ester mediated mitochondrial inhibition), suggesting the feasibility of interventions for neuronal atrophy.^352^

**Fig. 4:**
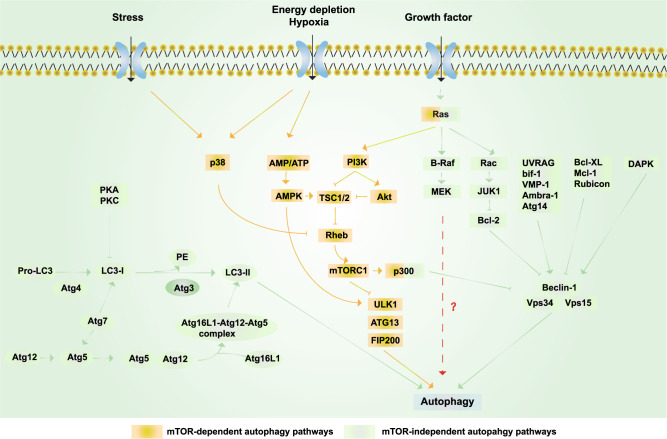
Schematic representation of autophagy. Yellow box: mTOR-dependent autophagy pathways. Growth factors can inhibit autophagy via activating the PI3K/Akt/mTORC1 pathway; under nutrient-rich conditions, mTORC1 is activated, whereas under starvation and oxidative stress, mTORC1 is inhibited. AMPK-dependent autophagy activation can be induced by starvation and hypoxia.^449^ Ras can also activate autophagy via activating PI3K,^352^ while p300 can inhibit autophagy.^450^ p38 promotes autophagy by phosphorylating and inactivating Rheb and then inhibiting mTOR under stress.^451^ Green boxes: mTOR-independent autophagy pathways. The PI3KCIII complex (also called the Beclin 1–Vps34–Vps15 complex) is essential for the induction of autophagy and is regulated by interacting proteins, such as the negative regulators Rubicon, Mcl-1, and Bcl-XL/Bcl-2, while proteins including UVRAG, Atg14, Bif-1, VMP-1, and Ambra-1 induce autophagy by binding Beclin 1 and Vps34 and promoting the activity of the PI3KCIII complex.^357^ In addition, various kinases also regulate autophagy. ERK and JNK-1 can phosphorylate Bcl-2, release its inhibition, and consequently induce autophagy; the phosphorylation of Beclin 1 by Akt inhibits autophagy, whereas the phosphorylation of Beclin 1 by DAPK promotes autophagy.^452^ Autophagy can be inhibited by the action of PKA and PKC on LC3. Finally, Atg4, Atg3, Atg7, and Atg10 are autophagy-related proteins that mediate the formation of the Atg12–Atg5–Atg16L1 complex and LC3-II.^453^ RAS and p300 can also regulate autophagy via the mTOR-independent pathway^454^

The MAPK pathway protects neurons against dendritic atrophy and relies on MEK-dependent autophagy.^352^ Autophagy is the principal cellular pathway by which degraded proteins and organelles are recycled, and it plays an essential role in cell fate in response to stress.^353–357^ Aged organelles and protein aggregates are cleared by the autophagosome–lysosome pathway, which is particularly important in neurons.^358–360^ Growing evidence has implicated defective autophagy in neurodegenerative diseases, including AD, PD, amyotrophic lateral sclerosis and HD.^358,361–364^ Recent work using live-cell imaging determined that autophagosomes preferentially form at the axon tip and undergo retrograde transport to the cell body.^365^ As a key protein in autophagy, Beclin 1 is decreased in the early stage of AD.^357,366,367^ Moreover, a decrease in autophagy induced by the genetic ablation of Beclin 1 increases intracellular Aβ accumulation, extracellular Aβ deposition, and neurodegeneration.^368^ Autophagy decline also causes microglial impairments and neuronal ultrastructural abnormalities.^368^ On the other hand, transcriptome evidence has revealed enhanced autophagy–lysosome function in centenarians.^369^ PPARA-mediated autophagy can reduce AD-like pathology and cognitive decline.^370^ These results suggest that autophagy is a potential therapeutic target for AD. MEK-dependent autophagy is protective in neuronal cells.^352^ The activation of the MEK–ERK signaling pathway can reduce the production of toxic amyloid Aβ by inhibiting γ-secretase activity.^371–375^ Thus, MEK-dependent autophagy may provide a potential way to enhance Aβ and NFT clearance and may also be a new potential target for AD therapy (Fig. 4).

**Fig. 5:**
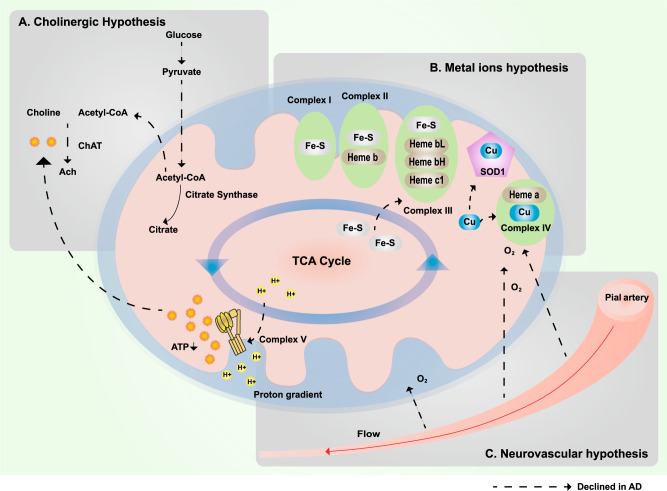
In addition to mitochondrial dysfunction, hypometabolism may underlie the cholinergic hypothesis, metal ion hypothesis, and neurovascular hypothesis. **a** Glucose is enzymatically catalyzed to produce pyruvate. Pyruvate is converted to acetyl-CoA and then enters the TCA cycle or is used in the cytoplasm to synthesize acetylcholine. However, in AD patients, because of hypometabolism, the production of acetyl-CoA and ATP is insufficient, which leads to a reduction in acetylcholine synthesis. **b** Mitochondrial complexes I–III require Fe/S clusters, and complexes II–IV need hemoproteins for electron transfer and the oxidative phosphorylation of the respiratory chain. When iron deficiency occurs, the production of Fe/S and hemoproteins decreases, thereby affecting mitochondrial function and resulting in hypometabolism. In addition, copper is essential for the function of complex IV. Clearly, Cu–Zn superoxide dismutase (SOD1) requires copper and zinc.^455,456^ **c** Hypoperfusion and hypoxia in vascular diseases leads to insufficient oxygen supply, which in turn leads to insufficient ATP synthesis, resulting in hypometabolism in AD patients. TCA: tricarboxylic acid cycle; SOD1: superoxide dismutase 1

Hypometabolism is sufficient to cause neuronal atrophy in vitro and in vivo.^352,376,377^ Hypometabolism may be a potential therapeutic target for AD.^378^ Regional hypometabolism is another characteristic of AD brains (Fig. 5). The human brain makes up ~2% of the body weight but consumes up to ~20% of the oxygen supply; the brain is energy demanding and relies on the efficiency of the mitochondrial TCA cycle and oxidative phosphorylation for ATP generation.^379–382^ However, glucose metabolism in the brain in AD and mild cognitive impairment is significantly impaired compared with that in the brain upon normal aging, and the decline in cerebral glucose metabolism occurs before pathology and symptoms manifest and gradually worsens as symptoms progress.^383–385^ In 1983, de Leon et al. examined aged patients with senile dementia and found a 17–24% decline in the cerebral glucose metabolic rate.^386^ Inefficient glucose utilization, impaired ATP production, and oxidative damage are closely correlated, and these deficiencies have profound consequences in AD.^387,388^ For example, ATP deficiency causes the loss of the neuronal membrane potential since Na^+^/K^+^ ATPase fails to maintain proper intracellular and extracellular gradients of Na^+^ and K^+^ ions. In addition, the propagation of action potentials and the production of neurotransmission is hindered by energy insufficiency. Moreover, after membrane depolarization (mainly due to the dissipation of Na^+^ and K^+^ ion gradients), Ca^2+^ flows down the steep gradient (~1.2 mM of extracellular Ca^2+^ to ~0.1 μM of intracellular Ca^2+^) into the cell to raise intracellular Ca^2+^ levels and stimulates the activities of various Ca^2+^-dependent enzymes (including endonucleases, phospholipases, and proteinases), eventually contributing to neuronal dysfunction and death.^158^ Mitochondria are the most energetically and metabolically active organelles in the cell.^389,390^ Mitochondria are also dynamic organelles; they experiences changes in their functional capacities, morphologies, and positions^391–393^ so that they can be transported, and they respond to physiological signals to meet the energy and metabolic demands of cellular activities.^394–396^ In addition to neuronal atrophy, mitochondrial dysfunction leads to hypometabolism, which in turn contributes to the progression of AD.^397–399^ Indeed, there is evidence that hypometabolism and neuronal atrophy coexists in patients with amyloid-negative AD.^400^ In addition to mitochondrial dysfunction, hypoperfusion and hypoxia in vascular diseases may also cause hypometabolism in the brain and thus contribute to the progression of AD (Fig. 5). Meanwhile, as the synthesis of acetylcholine requires the involvement of acetyl-CoA and ATP, hypometabolism leads to a decrease in acetylcholine synthesis in neurons, which suggests that hypometabolism may be an underlying explanation for the acetylcholine hypothesis (Fig. 5).

**Fig. 6:**
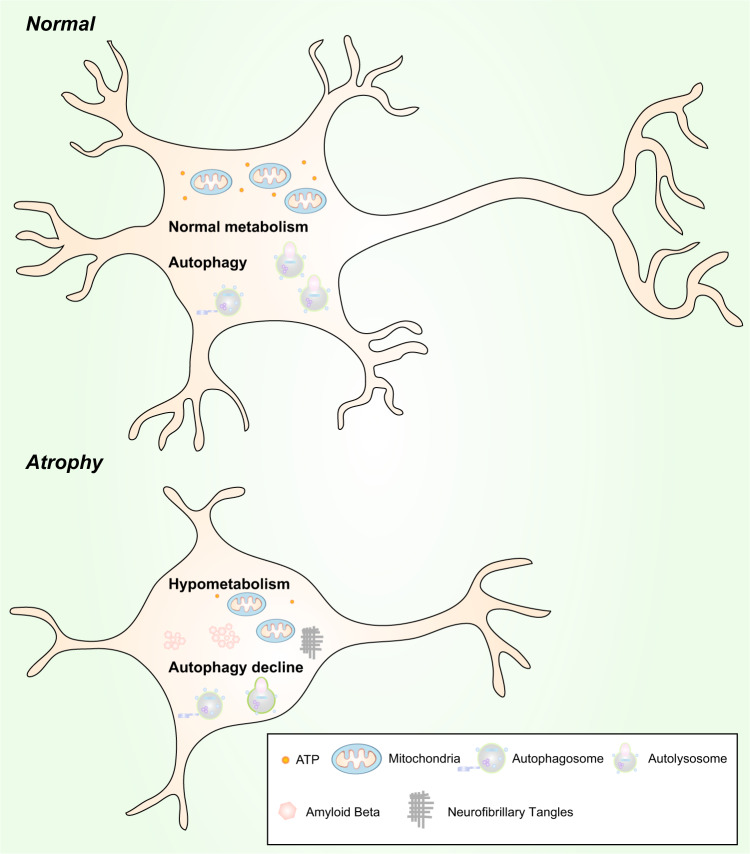
Hypometabolism and autophagy decline are likely to be causative factors of neuronal atrophy. Normal neurons vs. atrophic neurons. Upper: Normal levels of autophagy and metabolism exist in neurons to maintain their morphology and function. Lower: Hypometabolism and a reduction in autophagy are found in atrophic neurons

The relationship between hypometabolism and autophagy in neurons is still unknown,^352^ but calorie restriction (CR) is known to enhance autophagy. CR-induced autophagy can recycle intracellular degraded components and aggregates to maintain mitochondrial function.^401^ Hypometabolism and a simultaneous decrease in autophagy can worsen the situation and lead to the dysfunction and atrophy of neurons. Hypometabolism and a simultaneous decrease in autophagy may be causative factors of brain atrophy and AD (Fig. 6).

### Perspective

**Fig. 7:**
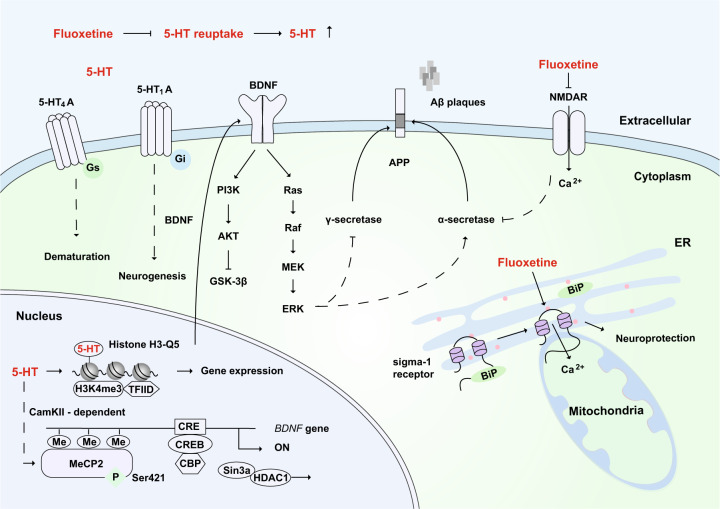
The potential mechanisms of fluoxetine in the remission of AD. As a selective 5-HT reuptake inhibitor, fluoxetine can increase the extraneuronal concentration of 5-HT. 5-HT binds to the 5-HT_4_A receptor to promote neuronal dematuration through a Gs-mediated pathway. 5-HT binds to the 5-HT_1_A receptor, which is involved in BDNF-dependent neurogenesis through the Gi-mediated signaling pathway. After 5-HT stimulation, MeCP2 is phosphorylated at Ser421 through CaMKII-dependent signaling, and this promotes the dissociation of CREB from HDAC and then increases the expression of BDNF. BDNF activates downstream signaling pathways, including the MEK-ERK pathway, which might promote the activity of α-secretase, inhibit γ-secretase, and reduce the production of toxic amyloid Aβ. Moreover, the serotonylation of histone H3 at glutamine 5 (Q5) enhances the binding of H3K4me3 and TFIID and allows gene expression. Fluoxetine has been reported to bind and inhibit NMDA receptors directly, which can reduce the inhibition of α-secretase and thus prevent the production of Aβ. In addition, fluoxetine can bind to the endoplasmic reticulum protein sigma-1 receptor, which induces the dissociation of Bip from the sigma-1 receptor and promotes neuroprotection. 5-HT: serotonin; ER: endoplasmic reticulum

AD, like the aging population, has increasingly become a medical and social concern. There are currently four clinically used drugs (a total of five therapies, the fifth one of which is a combination of two drugs) that have been approved by the FDA, but they only treat the symptoms and have no significant effect on the progression of AD. Based on this retrospective review of AD and the lessons learned, we propose that fluoxetine,^402^ a selective serotonin reuptake inhibitor (SSRI), may have strong potential for the treatment of AD (Fig. 7).

Based on functional brain imaging with PET, there is evidence that serotonin plays an important role in aging, late-life depression, and AD.^403^ Short-term treatment with the antidepressant fluoxetine can trigger pyramidal dendritic spine synapse formation in the rat hippocampus.^404^ In an MRI study of fluoxetine for the treatment of major depression, Vakili et al. found that female responders had a statistically significant higher mean right hippocampal volume than that of nonresponders.^405^ Long-term treatment with fluoxetine can promote the neurogenesis and proliferation of hippocampal neurons in mice through the 5-HT_1_A receptor, and this can relieve anxiety phenotypes in mice^406^ and enhance mitochondrial motility.^407^ 5-HT_4_A receptors that are expressed by mature neurons in the hippocampal dentate gyrus are also important for promoting neurogenesis and dematuration.^408–410^ Fluoxetine can promote neurogenesis not only in the hippocampus but also in the anterior cortex and hypothalamus.^411^ This action depends on BDNF, as fluoxetine can enhance the phosphorylation of methyl-CpG binding protein 2 (MeCP2) at serine 421 to relieve its transcriptional inhibition and thereby promote the expression of BDNF.^412,413^ In addition to promoting neurite outgrowth and neurogenesis, enhanced BDNF signaling can rearrange the subcellular distribution of α-secretase, which increases its binding to APP peptides; in addition, the activity of β-secretase is inhibited after BDNF treatment.^414^ Moreover, the serotonylation of glutamine (at position 5) in histone H3 by a transglutaminase 2-mediated manner is a sign of permissive gene expression.^415^

Furthermore, fluoxetine has been reported to bind and inhibit NMDA receptors directly in the CNS,^416^ and this can reduce the inhibition of α-secretase and thus prevent the production of Aβ.^203,417^ Fluoxetine also inhibits γ-secretase activity and reduces the production of toxic amyloid Aβ by activating MEK-ERK signaling.^371,372^ In addition, fluoxetine can bind to the endoplasmic reticulum protein sigma-1 receptor.^418^ Sigma-1 receptor ligands can enhance acetylcholine secretion.^419,420^ The sigma-1 receptor activator Anavex 2–73 has entered a phase III clinical trial after it was granted fast-track status by the FDA because of the promising results in phase II. The sigma-1 receptor is located in the mitochondrion-associated ER membrane so that the activation of the sigma-1 receptor can prolong Ca^2+^ signaling in mitochondria.^421^ Consequently, the local and specific elevation of [Ca^2+^] in the mitochondrial matrix can enhance ATP synthesis,^422,423^ which ameliorates hypometabolism.

In addition, our group examined the effect of SSRIs on cognitive function in AD by conducting a meta-analysis of randomized controlled studies. Of the 854 articles identified, 14 articles that involved 1091 participants were eligible for inclusion. We compared changes in MMSE scores between SSRI treatment groups and the placebo group, and we found that SSRIs may contribute to improved cognitive function, with a mean difference (MD) of 0.84 (95%CI: 0.32–1.37, *P* *=* 0.002) compared with the control. Further subgroup analysis exploring the effect of fluoxetine and other SSRIs revealed a beneficial effect of fluoxetine (MD = 1.16, 95%CI: 0.41–1.90, *P* *=* 0.002) but no benefit of other SSRIs (MD = 0.58, 95%CI: −0.17–1.33, *P* *=* 0.13) on cognitive function.^424^ Consequently, all of the above evidence indicates that fluoxetine has strong potential for the treatment of AD. In addition, because of above wealthy supporting documentation and the weak role of other SSRIs such as escitalopram in promoting BDNF release,^425^ fluoxetine was singled out as a potential therapy for the treatment of AD, not just as a complementary treatment.^426^ As summarized and illustrated in Fig. 7, the exact mechanisms of the effects of fluoxetine remain to be further clarified.

Finally, to summarize this review of the history and progress of hypotheses and clinical trials for AD, the most perplexing question is in regards to amyloid hypothesis and its failed clinical trials, which account for 22.3% of all clinical trials (Fig. 1). Although mutations in *APP*, *PSEN1*, or *PSEN2* only account for ~0.5% of all AD cases,^11^ mutations in PSEN1, which is the most common known genetic cause of FD and functions as the catalytic subunit of γ-secretase,^427,428^ may cast light upon Aβ and its paradox. In 2017, Sun et al. analyzed the effect of 138 pathogenic mutations in *PSEN1* on the production of Aβ−42 and Aβ−40 peptides by γ-secretase in vitro; they found that 90% of these mutations led to a decrease in the production of Aβ−42 and Aβ−40 and that 10% of these mutations result in decreased Aβ−42/Aβ−40 ratios.^429^ This comprehensive assessment of the impact of FD mutations on γ-secretase activity and Aβ production does not support the amyloid hypothesis and suggests an alternative therapeutic strategy aimed at restoring γ-secretase activity^430^; this is also supported by the fact that the functional loss of both *PSEN1* and *PSEN2* in the mouse postnatal forebrain causes memory impairment in an age-dependent manner.^431^ Considering that the activation of Notch signaling by the cleavage of γ-secretase^432^ is not involved in age-related neurodegeneration,^433^ other signaling pathways mediated by Aβ and/or other products of γ-secretase substrates, such as ErbB4,^434^ E-cadherin,^435^ N-cadherin,^436^ ephrin-B2,^437^ CD44,^438^ and LDL receptor-related protein,^439^ may play active roles in neuronal survival in the adult brain.

The most interesting and challenging phenomena regarding fluoxetine is that fluoxetine is clinically more effective in women than in men^440^ and that the prevalence of AD and other dementias is higher in women than in men^441^; meanwhile, women live significantly longer than men.^442^ These phenomena suggest that there are interplays or trade-offs between AD and longevity. In particular, *APOE* is the strongest genetic risk factor for AD^18–21^ and is the only gene associated with longevity that achieves genome-wide significance (*P* < 5 × 10^–8^).^443^ *APOE4* is associated with a risk of AD that declines after the age of 70; the OR for *APOE4* heterozygotes remains above unity at almost all ages; surprisingly, however, the OR for *APOE4* homozygotes dips below unity after the age of 89.^444^ There may be genetic and nongenetic factors that interact with *APOE4*, lead to shorter survival in more aggressive form of AD, or promote longevity in an age-dependent manner.^11^ Uncovering the puzzle of *APOE4* and the mystery of longevity may provide insights for AD prevention.

## References

[1] Blennow K, de Leon MJ, Zetterberg H (2006). Alzheimer’s disease. Lancet.

[2] Nelson PT, Braak H, Markesbery WR (2009). Neuropathology and cognitive impairment in Alzheimer disease: a complex but coherent relationship. J. Neuropathol. Exp. Neurol..

[3] Brookmeyer R, Gray S, Kawas C (1998). Projections of Alzheimer’s disease in the United States and the public health impact of delaying disease onset. Am. J. Public Health.

[4] Breteler MM, van den Ouweland FA, Grobbee DE, Hofman A (1992). A community-based study of dementia: the Rotterdam elderly study. Neuroepidemiology.

[5] Ferri CP (2005). Global prevalence of dementia: a Delphi consensus study. Lancet.

[6] Weiner MW (2012). The Alzheimer’s Disease Neuroimaging Initiative: a review of papers published since its inception. Alzheimers Dement..

[7] Launer LJ (1992). Overview of incidence studies of dementia conducted in Europe. Neuroepidemiology.

[8] Kidd M (1963). Paired helical filaments in electron microscopy of Alzheimer’s disease. Nature.

[9] Terry RD (1963). The fine structure of neurofibrillary tangles in Alzheimer’s disease. J. Neuropathol. Exp. Neurol..

[10] Hardy J (2006). A hundred years of Alzheimer’s disease research. Neuron.

[11] Ertekin-Taner N (2007). Genetics of Alzheimer’s disease: a centennial review. Neurol. Clin..

[12] Canter RG, Penney J, Tsai LH (2016). The road to restoring neural circuits for the treatment of Alzheimer’s disease. Nature.

[13] Reitz C, Mayeux R (2014). Alzheimer disease: epidemiology, diagnostic criteria, risk factors and biomarkers. Biochemical Pharmacol..

[14] Bateman RJ (2012). Clinical and biomarker changes in dominantly inherited Alzheimer’s disease. New Engl. J. Med..

[15] McInnes J (2013). Insights on altered mitochondrial function and dynamics in the pathogenesis of neurodegeneration. Transl. Neurodegener..

[16] Cuyvers E, Sleegers K (2016). Genetic variations underlying Alzheimer’s disease: evidence from genome-wide association studies and beyond. Lancet Neurol..

[17] Lambert JC (2013). Meta-analysis of 74,046 individuals identifies 11 new susceptibility loci for Alzheimer’s disease. Nat. Genet..

[18] Goate A (1991). Segregation of a missense mutation in the amyloid precursor protein gene with familial Alzheimer’s disease. Nature.

[19] Saunders AM (1993). Association of apolipoprotein E allele epsilon 4 with late-onset familial and sporadic Alzheimer’s disease. Neurology.

[20] Rogaev EI (1995). Familial Alzheimer’s disease in kindreds with missense mutations in a gene on chromosome 1 related to the Alzheimer’s disease type 3 gene. Nature.

[21] Sherrington R (1995). Cloning of a gene bearing missense mutations in early-onset familial Alzheimer’s disease. Nature.

[22] Corder EH (1993). Gene dose of apolipoprotein E type 4 allele and the risk of Alzheimer’s disease in late onset families. Science.

[23] Bu G (2009). Apolipoprotein E and its receptors in Alzheimer’s disease: pathways, pathogenesis and therapy. Nat. Rev. Neurosci..

[24] Farrer LA (1997). Effects of age, sex, and ethnicity on the association between apolipoprotein E genotype and Alzheimer disease. A meta-analysis. APOE and Alzheimer disease meta analysis consortium. JAMA.

[25] Liu CC, Liu CC, Kanekiyo T, Xu H, Bu G (2013). Apolipoprotein E and Alzheimer disease: risk, mechanisms and therapy. Nat. Rev. Neurol..

[26] Strittmatter WJ (1993). Apolipoprotein E: high-avidity binding to beta-amyloid and increased frequency of type 4 allele in late-onset familial Alzheimer disease. Proc. Natl Acad. Sci. USA.

[27] Poirier J (1993). Apolipoprotein E polymorphism and Alzheimer’s disease. Lancet.

[28] Ma J, Brewer HB, Potter H (1996). Alzheimer A beta neurotoxicity: promotion by antichymotrypsin, ApoE4; inhibition by A beta-related peptides. Neurobiol. Aging.

[29] Tiraboschi P (2004). Impact of APOE genotype on neuropathologic and neurochemical markers of Alzheimer disease. Neurology.

[30] Hoe HS (2009). Interaction of reelin with amyloid precursor protein promotes neurite outgrowth. J. Neurosci..

[31] Davies P, Maloney AJ (1976). Selective loss of central cholinergic neurons in Alzheimer’s disease. Lancet.

[32] Selkoe DJ (1991). The molecular pathology of Alzheimer’s disease. Neuron.

[33] Hardy J, Allsop D (1991). Amyloid deposition as the central event in the aetiology of Alzheimer’s disease. Trends Pharmacol. Sci..

[34] Frost B, Jacks RL, Diamond MI (2009). Propagation of tau misfolding from the outside to the inside of a cell. J. Biol. Chem..

[35] Swerdlow RH, Khan SM (2004). A “mitochondrial cascade hypothesis” for sporadic Alzheimer’s disease. Med. Hypotheses.

[36] Mattson MP (1992). beta-Amyloid peptides destabilize calcium homeostasis and render human cortical neurons vulnerable to excitotoxicity. J. Neurosci..

[37] McGeer PL, Rogers J (1992). Anti-inflammatory agents as a therapeutic approach to Alzheimer’s disease. Neurology.

[38] Iadecola C (2004). Neurovascular regulation in the normal brain and in Alzheimer’s disease. Nat. Rev. Neurosci..

[39] Bush AI (1994). Rapid induction of Alzheimer A beta amyloid formation by zinc. Science.

[40] Deane R (2004). LRP/amyloid beta-peptide interaction mediates differential brain efflux of Abeta isoforms. Neuron.

[41] Silverman JM (1994). The consortium to establish a registry for Alzheimer’s disease (CERAD). Part VI. Family history assessment: a multicenter study of first-degree relatives of Alzheimer’s disease probands and nondemented spouse controls. Neurology.

[42] Launer LJ (2000). Midlife blood pressure and dementia: the Honolulu-Asia aging study. Neurobiol. Aging.

[43] Osorio RS (2011). Greater risk of Alzheimer’s disease in older adults with insomnia. J. Am. Geriatrics Soc..

[44] Whitmer RA, Gunderson EP, Barrett-Connor E, Quesenberry CP, Yaffe K (2005). Obesity in middle age and future risk of dementia: a 27 year longitudinal population based study. BMJ.

[45] Butterfield DA, Halliwell B (2019). Oxidative stress, dysfunctional glucose metabolism and Alzheimer disease. Nat. Rev. Neurosci..

[46] Martins RN (2018). Alzheimer’s disease: a journey from amyloid peptides and oxidative stress, to biomarker technologies and disease prevention strategies-gains from AIBL and DIAN cohort. J. Alzheimers Dis..

[47] Mukherjee, S. et al. Genetic data and cognitively defined late-onset Alzheimer’s disease subgroups. *Mol. Psychiatry*, 10.1038/s41380-018-0298-8 (2018). 10.1038/s41380-018-0298-8 PMC6548676 30514930

[48] Aupperle PM (2006). Navigating patients and caregivers through the course of Alzheimer’s disease. J. Clin. Psychiatry.

[49] Qian X, Hamad B, Dias-Lalcaca G (2015). The Alzheimer disease market. Nat. Rev. Drug Discov..

[50] Francis PT, Palmer AM, Snape M, Wilcock GK (1999). The cholinergic hypothesis of Alzheimer’s disease: a review of progress. J. Neurol. Neurosurg. Psychiatry.

[51] Fotiou D, Kaltsatou A, Tsiptsios D, Nakou M (2015). Evaluation of the cholinergic hypothesis in Alzheimer’s disease with neuropsychological methods. Aging Clin. Exp. Res..

[52] Ferreira-Vieira TH, Guimaraes IM, Silva FR, Ribeiro FM (2016). Alzheimer’s disease: targeting the cholinergic system. Curr. Neuropharmacol..

[53] Bowen DM, Smith CB, White P, Davison AN (1976). Neurotransmitter-related enzymes and indices of hypoxia in senile dementia and other abiotrophies. Brain.

[54] White P (1977). Neocortical cholinergic neurons in elderly people. Lancet.

[55] Perry EK, Perry RH, Blessed G, Tomlinson BE (1977). Necropsy evidence of central cholinergic deficits in senile dementia. Lancet.

[56] Hakansson L (1993). Mechanism of action of cholinesterase inhibitors in Alzheimer’s disease. Acta Neurol. Scand. Suppl..

[57] Anand P, Singh B (2013). A review on cholinesterase inhibitors for Alzheimer’s disease. Arch. Pharmacal Res..

[58] O'Regan J, Lanctot KL, Mazereeuw G, Herrmann N (2015). Cholinesterase inhibitor discontinuation in patients with Alzheimer’s disease: a meta-analysis of randomized controlled trials. J. Clin. Psychiatry.

[59] Deardorff WJ, Feen E, Grossberg GT (2015). The use of cholinesterase inhibitors across all stages of Alzheimer’s disease. Drugs Aging.

[60] Davis KL, Powchik P (1995). Tacrine. Lancet.

[61] Bullock R (2005). Rivastigmine and donepezil treatment in moderate to moderately-severe Alzheimer’s disease over a 2-year period. Curr. Med. Res. Opin..

[62] Wilcock G (2003). A long-term comparison of galantamine and donepezil in the treatment of Alzheimer’s disease. Drugs aging.

[63] Mintzer JE, Kershaw P (2003). The efficacy of galantamine in the treatment of Alzheimer’s disease: comparison of patients previously treated with acetylcholinesterase inhibitors to patients with no prior exposure. Int. J. Geriatr. psychiatry.

[64] Knight R, Khondoker M, Magill N, Stewart R, Landau S (2018). A systematic review and meta-analysis of the effectiveness of acetylcholinesterase inhibitors and memantine in treating the cognitive symptoms of dementia. Dement. Geriatr. Cogn. Disord..

[65] Lon, S. S. (2000). A critical review of cholinesterase inhibitors as a treatment modality in Alzheimer’s disease. Dialogues Clin. Neurosci..

[66] Dementia – caring, ethics, ethnical and economical aspects: a systematic review. Stockholm: Swedish Council on Health Technology Assessment (SBU). SBU No. 172 (2008). 28876770

[67] Drugs for Alzheimer’s disease: best avoided. No therapeutic advantage. *Prescrire Int.* **21**, 150 (2012). 22822592

[68] Hardy J, Selkoe DJ (2002). The amyloid hypothesis of Alzheimer’s disease: progress and problems on the road to therapeutics. Science.

[69] Quon D (1991). Formation of beta-amyloid protein deposits in brains of transgenic mice. Nature.

[70] Bennett DA (2006). Neuropathology of older persons without cognitive impairment from two community-based studies. Neurology.

[71] De Meyer G (2010). Diagnosis-independent Alzheimer disease biomarker signature in cognitively normal elderly people. Arch. Neurol..

[72] Fagan AM (2007). Cerebrospinal fluid tau/beta-amyloid(42) ratio as a prediction of cognitive decline in nondemented older adults. Arch. Neurol..

[73] Gomperts SN (2008). Imaging amyloid deposition in Lewy body diseases. Neurology.

[74] Glenner GG, Wong CW (1984). Alzheimer’s disease: initial report of the purification and characterization of a novel cerebrovascular amyloid protein. Biochemical Biophysical Res. Commun..

[75] Kang J (1987). The precursor of Alzheimer’s disease amyloid A4 protein resembles a cell-surface receptor. Nature.

[76] St George-Hyslop PH (1987). The genetic defect causing familial Alzheimer’s disease maps on chromosome 21. Science.

[77] Mullan M (1992). A pathogenic mutation for probable Alzheimer’s disease in the APP gene at the N-terminus of beta-amyloid. Nat. Genet..

[78] Di Fede G (2009). A recessive mutation in the APP gene with dominant-negative effect on amyloidogenesis. Science.

[79] Karran E, Mercken M, De Strooper B (2011). The amyloid cascade hypothesis for Alzheimer’s disease: an appraisal for the development of therapeutics. Nat. Rev. Drug Discov..

[80] Sisodia SS, Koo EH, Beyreuther K, Unterbeck A, Price DL (1990). Evidence that beta-amyloid protein in Alzheimer’s disease is not derived by normal processing. Science.

[81] Jonsson T (2012). A mutation in APP protects against Alzheimer’s disease and age-related cognitive decline. Nature.

[82] Yankner BA, Duffy LK, Kirschner DA (1990). Neurotrophic and neurotoxic effects of amyloid beta protein: reversal by tachykinin neuropeptides. Science.

[83] Wang J, Dickson DW, Trojanowski JQ, Lee VM (1999). The levels of soluble versus insoluble brain Abeta distinguish Alzheimer’s disease from normal and pathologic aging. Exp. Neurol..

[84] Walsh DM (2002). Naturally secreted oligomers of amyloid beta protein potently inhibit hippocampal long-term potentiation in vivo. Nature.

[85] Lewis J (2001). Enhanced neurofibrillary degeneration in transgenic mice expressing mutant tau and APP. Science.

[86] Palop JJ (2007). Aberrant excitatory neuronal activity and compensatory remodeling of inhibitory hippocampal circuits in mouse models of Alzheimer’s disease. Neuron.

[87] Lim HK (2014). Regional amyloid burden and intrinsic connectivity networks in cognitively normal elderly subjects. Brain.

[88] Lim YY (2014). Effect of amyloid on memory and non-memory decline from preclinical to clinical Alzheimer’s disease. Brain.

[89] Lim YY (2014). Abeta and cognitive change: examining the preclinical and prodromal stages of Alzheimer’s disease. Alzheimer’s Dement..

[90] Knopman DS (2012). Short-term clinical outcomes for stages of NIA-AA preclinical Alzheimer disease. Neurology.

[91] Vos SJ (2013). Preclinical Alzheimer’s disease and its outcome: a longitudinal cohort study. Lancet Neurol..

[92] Golde TE (2014). Open questions for Alzheimer’s disease immunotherapy. Alzheimer’s. Res. Ther..

[93] Honig LS (2018). Trial of Solanezumab for Mild Dementia Due to Alzheimer’s Disease. New Engl. J. Med..

[94] Ostrowitzki S (2017). A phase III randomized trial of gantenerumab in prodromal Alzheimer’s disease. Alzheimer’s. Res. Ther..

[95] Egan MF (2018). Randomized trial of verubecestat for mild-to-moderate Alzheimer’s disease. New Engl. J. Med..

[96] Doody RS (2013). A phase 3 trial of semagacestat for treatment of Alzheimer’s disease. New Engl. J. Med..

[97] Wolfe MS (2008). Inhibition and modulation of gamma-secretase for Alzheimer’s disease. Neurotherapeutics.

[98] Braak H, Braak E (1996). Evolution of the neuropathology of Alzheimer’s disease. Acta Neurol. Scand. Suppl..

[99] Braak E (1999). Neuropathology of Alzheimer’s disease: what is new since A. Alzheimer?. Eur. Arch. Psychiatry Clin. Neurosci..

[100] Nukina N, Ihara Y (1986). One of the antigenic determinants of paired helical filaments is related to tau protein. J. Biochem..

[101] Grundke-Iqbal I (1986). Microtubule-associated protein tau. A component of Alzheimer paired helical filaments. J. Biol. Chem..

[102] Kosik KS, Joachim CL, Selkoe DJ (1986). Microtubule-associated protein tau (tau) is a major antigenic component of paired helical filaments in Alzheimer disease. Proc. Natl Acad. Sci. USA.

[103] Grundke-Iqbal I (1986). Abnormal phosphorylation of the microtubule-associated protein tau (tau) in Alzheimer cytoskeletal pathology. Proc. Natl Acad. Sci. USA.

[104] Clavaguera F (2009). Transmission and spreading of tauopathy in transgenic mouse brain. Nat. Cell Biol..

[105] Goedert M, Wischik CM, Crowther RA, Walker JE, Klug A (1988). Cloning and sequencing of the cDNA encoding a core protein of the paired helical filament of Alzheimer disease: identification as the microtubule-associated protein tau. Proc. Natl Acad. Sci. USA.

[106] Lee G, Cowan N, Kirschner M (1988). The primary structure and heterogeneity of tau protein from mouse brain. Science.

[107] Goedert M, Spillantini MG, Jakes R, Rutherford D, Crowther RA (1989). Multiple isoforms of human microtubule-associated protein tau: sequences and localization in neurofibrillary tangles of Alzheimer’s disease. Neuron.

[108] Andreadis A, Brown WM, Kosik KS (1992). Structure and novel exons of the human tau gene. Biochemistry.

[109] Adams SJ, DeTure MA, McBride M, Dickson DW, Petrucelli L (2010). Three repeat isoforms of tau inhibit assembly of four repeat tau filaments. PloS ONE.

[110] Allen B (2002). Abundant tau filaments and nonapoptotic neurodegeneration in transgenic mice expressing human P301S tau protein. J. Neurosci..

[111] Probst A (2000). Axonopathy and amyotrophy in mice transgenic for human four-repeat tau protein. Acta Neuropathol..

[112] Merrick SE, Trojanowski JQ, Lee VM (1997). Selective destruction of stable microtubules and axons by inhibitors of protein serine/threonine phosphatases in cultured human neurons. J. Neurosci..

[113] Buee L, Bussiere T, Buee-Scherrer V, Delacourte A, Hof PR (2000). Tau protein isoforms, phosphorylation and role in neurodegenerative disorders. Brain Res. Brain Res. Rev..

[114] Liu F, Iqbal K, Grundke-Iqbal I, Hart GW, Gong CX (2004). O-GlcNAcylation regulates phosphorylation of tau: a mechanism involved in Alzheimer’s disease. Proc. Natl Acad. Sci. USA.

[115] Lefebvre T (2003). Evidence of a balance between phosphorylation and O-GlcNAc glycosylation of Tau proteins-a role in nuclear localization. Biochimica et. Biophysica Acta.

[116] Lee G (2004). Phosphorylation of tau by fyn: implications for Alzheimer’s disease. J. Neurosci..

[117] Gong CX, Liu F, Grundke-Iqbal I, Iqbal K (2005). Post-translational modifications of tau protein in Alzheimer's disease. J. Neural Transm..

[118] Arriagada PV, Marzloff K, Hyman BT (1992). Distribution of Alzheimer-type pathologic changes in nondemented elderly individuals matches the pattern in Alzheimer’s disease. Neurology.

[119] Wittmann CW (2001). Tauopathy in Drosophila: neurodegeneration without neurofibrillary tangles. Science.

[120] Pastor P (2003). Apolipoprotein Eepsilon4 modifies Alzheimer’s disease onset in an E280A PS1 kindred. Ann. Neurol..

[121] Bales KR (2002). amyloid, and Alzheimer disease. Mol. Interventions.

[122] DeMattos RB (2004). ApoE and clusterin cooperatively suppress Abeta levels and deposition: evidence that ApoE regulates extracellular Abeta metabolism in vivo. Neuron.

[123] Fagan AM (2002). Human and murine ApoE markedly alters A beta metabolism before and after plaque formation in a mouse model of Alzheimer’s disease. Neurobiol. Dis..

[124] Brecht WJ (2004). Neuron-specific apolipoprotein e4 proteolysis is associated with increased tau phosphorylation in brains of transgenic mice. J. Neurosci..

[125] Harris FM (2003). Carboxyl-terminal-truncated apolipoprotein E4 causes Alzheimer’s disease-like neurodegeneration and behavioral deficits in transgenic mice. Proc. Natl Acad. Sci. USA.

[126] Gibb GM (2000). Differential effects of apolipoprotein E isoforms on phosphorylation at specific sites on tau by glycogen synthase kinase-3 beta identified by nano-electrospray mass spectrometry. FEBS Lett..

[127] Phiel CJ, Wilson CA, Lee VM, Klein PS (2003). GSK-3alpha regulates production of Alzheimer’s disease amyloid-beta peptides. Nature.

[128] Su Y (2004). Lithium, a common drug for bipolar disorder treatment, regulates amyloid-beta precursor protein processing. Biochemistry.

[129] Rapoport M, Dawson HN, Binder LI, Vitek MP, Ferreira A (2002). Tau is essential to beta -amyloid-induced neurotoxicity. Proc. Natl Acad. Sci. USA.

[130] Gong Li, Tian Xiangzhu, Zhou Jing, Dong Qiong, Tan Yan, Lu You, Wu Jiayan, Zhao Yanxin, Liu Xueyuan (2019). Iron Dyshomeostasis Induces Binding of APP to BACE1 for Amyloid Pathology, and Impairs APP/Fpn1 Complex in Microglia: Implication in Pathogenesis of Cerebral Microbleeds. Cell Transplantation.

[131] Xian-hui D (2015). Age-related changes of brain iron load changes in the frontal cortex in APPswe/PS1DeltaE9 transgenic mouse model of Alzheimer's disease.. J. Trace Elements Med. Biol..

[132] Li X (2015). Enduring elevations of hippocampal amyloid precursor protein and iron are features of beta-amyloid toxicity and are mediated by tau. Neurotherapeutics.

[133] Tuo QZ (2017). Tau-mediated iron export prevents ferroptotic damage after ischemic stroke. Mol. Psychiatry.

[134] Lei P (2012). Tau deficiency induces parkinsonism with dementia by impairing APP-mediated iron export. Nat. Med..

[135] Boutajangout A, Ingadottir J, Davies P, Sigurdsson EM (2011). Passive immunization targeting pathological phospho-tau protein in a mouse model reduces functional decline and clears tau aggregates from the brain. J. Neurochem..

[136] Asuni AA, Boutajangout A, Quartermain D, Sigurdsson EM (2007). Immunotherapy targeting pathological tau conformers in a tangle mouse model reduces brain pathology with associated functional improvements. J. Neurosci..

[137] Wilcock GK (2018). Potential of Low dose leuco-methylthioninium bis(hydromethanesulphonate) (LMTM) monotherapy for treatment of mild Alzheimer’s disease: cohort analysis as modified primary outcome in a phase III clinical trial. J. Alzheimer’s. Dis..

[138] Novak P (2018). FUNDAMANT: an interventional 72-week phase 1 follow-up study of AADvac1, an active immunotherapy against tau protein pathology in Alzheimer’s disease. Alzheimer’s. Res. Ther..

[139] Trifunovic A (2004). Premature ageing in mice expressing defective mitochondrial DNA polymerase. Nature.

[140] Kujoth GC (2005). Mitochondrial DNA mutations, oxidative stress, and apoptosis in mammalian aging. Science.

[141] Ross JM (2013). Germline mitochondrial DNA mutations aggravate ageing and can impair brain development. Nature.

[142] Jones DP (2006). Redefining oxidative stress. Antioxid. Redox Signal..

[143] Hauptmann N, Grimsby J, Shih JC, Cadenas E (1996). The metabolism of tyramine by monoamine oxidase A/B causes oxidative damage to mitochondrial DNA. Arch. Biochem. Biophys..

[144] Di Meo S, Reed TT, Venditti P, Victor VM (2016). Role of ROS and RNS sources in physiological and pathological conditions. Oxid. Med. Cell. Longev..

[145] Hirai K (2001). Mitochondrial abnormalities in Alzheimer’s disease. J. Neurosci..

[146] Ray PD, Huang BW, Tsuji Y (2012). Reactive oxygen species (ROS) homeostasis and redox regulation in cellular signaling. Cell. Signal..

[147] Holmstrom KM, Finkel T (2014). Cellular mechanisms and physiological consequences of redox-dependent signalling. Nat. Rev. Mol. Cell Biol..

[148] Uttara B, Singh AV, Zamboni P, Mahajan RT (2009). Oxidative stress and neurodegenerative diseases: a review of upstream and downstream antioxidant therapeutic options. Curr. Neuropharmacol..

[149] Gibson GE, Sheu KF, Blass JP (1998). Abnormalities of mitochondrial enzymes in Alzheimer disease. J. Neural Transm..

[150] Chandrasekaran K (1994). Impairment in mitochondrial cytochrome oxidase gene expression in Alzheimer disease. Brain Res. Mol. Brain Res..

[151] Cottrell DA, Blakely EL, Johnson MA, Ince PG, Turnbull DM (2001). Mitochondrial enzyme-deficient hippocampal neurons and choroidal cells in AD. Neurology.

[152] Maurer I, Zierz S, Moller HJ (2000). A selective defect of cytochrome c oxidase is present in brain of Alzheimer disease patients. Neurobiol. Aging.

[153] Nagy Z, Esiri MM, LeGris M, Matthews PM (1999). Mitochondrial enzyme expression in the hippocampus in relation to Alzheimer-type pathology. Acta Neuropathol..

[154] Bubber P, Haroutunian V, Fisch G, Blass JP, Gibson GE (2005). Mitochondrial abnormalities in Alzheimer brain: mechanistic implications. Ann. Neurol..

[155] Manczak M (2006). Mitochondria are a direct site of A beta accumulation in Alzheimer’s disease neurons: implications for free radical generation and oxidative damage in disease progression. Hum. Mol. Genet..

[156] Kamat PK (2016). Mechanism of oxidative stress and synapse dysfunction in the pathogenesis of Alzheimer’s disease: understanding the therapeutics strategies. Mol. Neurobiol..

[157] Snyder EM (2005). Regulation of NMDA receptor trafficking by amyloid-beta. Nat. Neurosci..

[158] Bezprozvanny I, Mattson MP (2008). Neuronal calcium mishandling and the pathogenesis of Alzheimer’s disease. Trends Neurosci..

[159] Gatz M (2006). Role of genes and environments for explaining Alzheimer disease. Arch. Gen. Psychiatry.

[160] Lunnon K, Mill J (2013). Epigenetic studies in Alzheimer’s disease: current findings, caveats, and considerations for future studies. Am. J. Med. Genet. B Neuropsychiatr. Genet..

[161] Gjoneska E (2015). Conserved epigenomic signals in mice and humans reveal immune basis of Alzheimer’s disease. Nature.

[162] Nativio R (2018). Publisher Correction: Dysregulation of the epigenetic landscape of normal aging in Alzheimer’s disease. Nat. Neurosci..

[163] Zhang K (2012). Targeted proteomics for quantification of histone acetylation in Alzheimer’s disease. Proteomics.

[164] Matilainen O, Quiros PM, Auwerx J (2017). Mitochondria and epigenetics: crosstalk in homeostasis and stress. Trends Cell Biol..

[165] Mastroeni D, McKee A, Grover A, Rogers J, Coleman PD (2009). Epigenetic differences in cortical neurons from a pair of monozygotic twins discordant for Alzheimer’s disease. PloS ONE.

[166] Mastroeni D (2010). Epigenetic changes in Alzheimer’s disease: decrements in DNA methylation. Neurobiol. Aging.

[167] Chouliaras L (2013). Consistent decrease in global DNA methylation and hydroxymethylation in the hippocampus of Alzheimer’s disease patients. Neurobiol. Aging.

[168] Condliffe D (2014). Cross-region reduction in 5-hydroxymethylcytosine in Alzheimer’s disease brain. Neurobiol. Aging.

[169] Teperino R, Schoonjans K, Auwerx J (2010). Histone methyl transferases and demethylases; can they link metabolism and transcription?. Cell Metab..

[170] Chiang PK (1996). S-Adenosylmethionine and methylation. FASEB J..

[171] Figueroa ME (2010). Leukemic IDH1 and IDH2 mutations result in a hypermethylation phenotype, disrupt TET2 function, and impair hematopoietic differentiation. Cancer Cell.

[172] Yan H (2009). IDH1 and IDH2 mutations in gliomas. New Engl. J. Med..

[173] Katewa SD, Khanna A, Kapahi P (2014). Mitobolites: the elixir of life. Cell Metab..

[174] Sciacovelli M (2016). Fumarate is an epigenetic modifier that elicits epithelial-to-mesenchymal transition. Nature.

[175] Hino S (2012). FAD-dependent lysine-specific demethylase-1 regulates cellular energy expenditure. Nat. Commun..

[176] Wellen KE (2009). ATP-citrate lyase links cellular metabolism to histone acetylation. Science.

[177] Imai S, Armstrong CM, Kaeberlein M, Guarente L (2000). Transcriptional silencing and longevity protein Sir2 is an NAD-dependent histone deacetylase. Nature.

[178] Fang EF (2019). Mitophagy and NAD(+) inhibit Alzheimer disease. Autophagy.

[179] Lemasters JJ (2005). Selective mitochondrial autophagy, or mitophagy, as a targeted defense against oxidative stress, mitochondrial dysfunction, and aging. Rejuvenation Res..

[180] Ryan BJ, Hoek S, Fon EA, Wade-Martins R (2015). Mitochondrial dysfunction and mitophagy in Parkinson's: from familial to sporadic disease. Trends Biochem. Sci..

[181] Khalil B (2015). PINK1-induced mitophagy promotes neuroprotection in Huntington's disease. Cell Death Dis..

[182] Sun N, Youle RJ, Finkel T (2016). The Mitochondrial Basis of Aging. Mol. Cell.

[183] Wong YC, Holzbaur EL (2014). Optineurin is an autophagy receptor for damaged mitochondria in parkin-mediated mitophagy that is disrupted by an ALS-linked mutation. Proc. Natl Acad. Sci. USA.

[184] Fang EF (2019). Mitophagy inhibits amyloid-beta and tau pathology and reverses cognitive deficits in models of Alzheimer’s disease. Nat. Neurosci..

[185] Kerr JS (2017). Mitophagy and Alzheimer’s Disease: Cellular and Molecular Mechanisms. Trends Neurosci..

[186] Lucin KM (2013). Microglial beclin 1 regulates retromer trafficking and phagocytosis and is impaired in Alzheimer’s disease. Neuron.

[187] Chin RM (2014). The metabolite alpha-ketoglutarate extends lifespan by inhibiting ATP synthase and TOR. Nature.

[188] Mouchiroud L (2011). Pyruvate imbalance mediates metabolic reprogramming and mimics lifespan extension by dietary restriction in Caenorhabditis elegans. Aging Cell.

[189] Williams DS, Cash A, Hamadani L, Diemer T (2009). Oxaloacetate supplementation increases lifespan in Caenorhabditis elegans through an AMPK/FOXO-dependent pathway. Aging Cell.

[190] Wilkins HM (2016). Oxaloacetate enhances neuronal cell bioenergetic fluxes and infrastructure. J. Neurochem..

[191] Wilkins HM (2014). Oxaloacetate activates brain mitochondrial biogenesis, enhances the insulin pathway, reduces inflammation and stimulates neurogenesis. Hum. Mol. Genet..

[192] Swerdlow RH, Bothwell R, Hutfles L, Burns JM, Reed GA (2016). Tolerability and pharmacokinetics of oxaloacetate 100 mg capsules in Alzheimer’s subjects. BBA Clin..

[193] Khachaturian ZS (1989). Calcium, membranes, aging, and Alzheimer’s disease. Introduction and overview. Ann. New Y. Acad. Sci..

[194] Marx J (2007). Alzheimer’s disease. Fresh evidence points to an old suspect: calcium. Science.

[195] Bezprozvanny I (2009). Calcium signaling and neurodegenerative diseases. Trends Mol. Med..

[196] Demuro A, Parker I, Stutzmann GE (2010). Calcium signaling and amyloid toxicity in Alzheimer disease. J. Biol. Chem..

[197] Norris CM (2005). Calcineurin triggers reactive/inflammatory processes in astrocytes and is upregulated in aging and Alzheimer’s models. J. Neurosci..

[198] Abdul HM (2009). Cognitive decline in Alzheimer’s disease is associated with selective changes in calcineurin/NFAT signaling. J. Neurosci..

[199] Berridge MJ (2013). Dysregulation of neural calcium signaling in Alzheimer disease, bipolar disorder and schizophrenia. Prion.

[200] FDA approves memantine drug for treating AD. *Am. J. Alzheimer’s. Dis. Other Dement*. **18**, 329–330 (2003). 14682078

[201] Reisberg B (2003). Memantine in moderate-to-severe Alzheimer’s disease. New Engl. J. Med..

[202] Johnson JW, Kotermanski SE (2006). Mechanism of action of memantine. Curr. Opin. Pharmacol..

[203] Lesne S (2005). NMDA receptor activation inhibits alpha-secretase and promotes neuronal amyloid-beta production. J. Neurosci..

[204] Zlokovic BV (2008). The blood-brain barrier in health and chronic neurodegenerative disorders. Neuron.

[205] Moskowitz MA, Lo EH, Iadecola C (2010). The science of stroke: mechanisms in search of treatments. Neuron.

[206] Guo S, Lo EH (2009). Dysfunctional cell-cell signaling in the neurovascular unit as a paradigm for central nervous system disease. Stroke.

[207] Buee L (1994). Pathological alterations of the cerebral microvasculature in Alzheimer’s disease and related dementing disorders. Acta Neuropathol.

[208] Thomas T, Thomas G, McLendon C, Sutton T, Mullan M (1996). beta-Amyloid-mediated vasoactivity and vascular endothelial damage. Nature.

[209] Iadecola C (1999). SOD1 rescues cerebral endothelial dysfunction in mice overexpressing amyloid precursor protein. Nat. Neurosci..

[210] Niwa K (2000). Abeta 1-40-related reduction in functional hyperemia in mouse neocortex during somatosensory activation. Proc. Natl Acad. Sci. USA.

[211] Ruitenberg A (2005). Cerebral hypoperfusion and clinical onset of dementia: the Rotterdam study. Ann. Neurol..

[212] Knopman DS, Roberts R (2010). Vascular risk factors: imaging and neuropathologic correlates. J. Alzheimer’s. Dis..

[213] Smith CD (1999). Altered brain activation in cognitively intact individuals at high risk for Alzheimer’s disease. Neurology.

[214] Bookheimer SY (2000). Patterns of brain activation in people at risk for Alzheimer's disease. N. Eng. J. Med..

[215] Zlokovic BV (2005). Neurovascular mechanisms of Alzheimer’s neurodegeneration. Trends Neurosci..

[216] Proitsi P (2014). Genetic predisposition to increased blood cholesterol and triglyceride lipid levels and risk of Alzheimer disease: a Mendelian randomization analysis. PLoS Med..

[217] Hassing LB (2009). Overweight in midlife and risk of dementia: a 40-year follow-up study. Int. J. Obes..

[218] Anstey KJ, Cherbuin N, Budge M, Young J (2011). Body mass index in midlife and late-life as a risk factor for dementia: a meta-analysis of prospective studies. Obes. Rev..

[219] Christensen A, Pike CJ (2015). Menopause, obesity and inflammation: interactive risk factors for Alzheimer’s disease. Front. Aging Neurosci..

[220] Letra L, Santana I, Seica R (2014). Obesity as a risk factor for Alzheimer’s disease: the role of adipocytokines. Metab. Brain Dis..

[221] Butterfield DA, Di Domenico F, Barone E (2014). Elevated risk of type 2 diabetes for development of Alzheimer disease: a key role for oxidative stress in brain. Biochimica et. Biophysica Acta.

[222] Biessels GJ, Strachan MW, Visseren FL, Kappelle LJ, Whitmer RA (2014). Dementia and cognitive decline in type 2 diabetes and prediabetic stages: towards targeted interventions.. Lancet.

[223] Moreira PI (2013). High-sugar diets, type 2 diabetes and Alzheimer’s disease. Curr. Opin. Clin. Nutr. Metab. care.

[224] Correia SC (2012). Insulin signaling, glucose metabolism and mitochondria: major players in Alzheimer’s disease and diabetes interrelation. Brain Res..

[225] Moreira PI (2012). Alzheimer’s disease and diabetes: an integrative view of the role of mitochondria, oxidative stress, and insulin. J. Alzheimer’s. Dis..

[226] Li G (2017). Effect of simvastatin on CSF Alzheimer disease biomarkers in cognitively normal adults. Neurology.

[227] Gold M (2010). Rosiglitazone monotherapy in mild-to-moderate Alzheimer’s disease: results from a randomized, double-blind, placebo-controlled phase III study. Dement. Geriatr. Cogn. Disord..

[228] Moonga I, Niccolini F, Wilson H, Pagano G, Politis M (2017). Hypertension is associated with worse cognitive function and hippocampal hypometabolism in Alzheimer’s disease. Eur. J. Neurol..

[229] Wharton W (2012). The effects of ramipril in individuals at risk for Alzheimer’s disease: results of a pilot clinical trial. J. Alzheimer’s. Dis..

[230] Bagyinszky E (2017). Role of inflammatory molecules in the Alzheimer’s disease progression and diagnosis. J. Neurological Sci..

[231] Latta CH, Brothers HM, Wilcock DM (2015). Neuroinflammation in Alzheimer’s disease; A source of heterogeneity and target for personalized therapy. Neuroscience.

[232] Phillips EC (2014). Astrocytes and neuroinflammation in Alzheimer’s disease. Biochemical Soc. Trans..

[233] Santos LE, Beckman D, Ferreira ST (2016). Microglial dysfunction connects depression and Alzheimer’s disease. Brain Behav. Immun..

[234] McGeer PL, Itagaki S, McGeer EG (1988). Expression of the histocompatibility glycoprotein HLA-DR in neurological disease. Acta Neuropathol.

[235] McGeer EG, McGeer PL (2010). Neuroinflammation in Alzheimer’s disease and mild cognitive impairment: a field in its infancy. J. Alzheimer’s. Dis..

[236] Meda L (1995). Activation of microglial cells by beta-amyloid protein and interferon-gamma. Nature.

[237] El Khoury J (1996). Scavenger receptor-mediated adhesion of microglia to beta-amyloid fibrils. Nature.

[238] Weldon DT (1998). Fibrillar beta-amyloid induces microglial phagocytosis, expression of inducible nitric oxide synthase, and loss of a select population of neurons in the rat CNS in vivo. J. Neurosci..

[239] Eikelenboom P, Stam FC (1982). Immunoglobulins and complement factors in senile plaques. An immunoperoxidase study. Acta Neuropathol..

[240] Michaud M (2013). Proinflammatory cytokines, aging, and age-related diseases. J. Am. Med. Dir. Assoc..

[241] Pluvinage JV (2019). CD22 blockade restores homeostatic microglial phagocytosis in ageing brains. Nature.

[242] Lyketsos CG (2007). Naproxen and celecoxib do not prevent AD in early results from a randomized controlled trial. Neurology.

[243] Qian X, Xu Z (2015). Fluorescence imaging of metal ions implicated in diseases. Chem. Soc. Rev..

[244] Scott LE, Orvig C (2009). Medicinal inorganic chemistry approaches to passivation and removal of aberrant metal ions in disease. Chem. Rev..

[245] Que EL, Domaille DW, Chang CJ (2008). Metals in neurobiology: probing their chemistry and biology with molecular imaging. Chem. Rev..

[246] Santner A, Uversky VN (2010). Metalloproteomics and metal toxicology of alpha-synuclein. Metallomics.

[247] Tamano H, Takeda A (2011). Dynamic action of neurometals at the synapse. Metallomics.

[248] Clements A, Allsop D, Walsh DM, Williams CH (1996). Aggregation and metal-binding properties of mutant forms of the amyloid A beta peptide of Alzheimer’s disease. J. Neurochem..

[249] Duce JA, Bush AI (2010). Biological metals and Alzheimer’s disease: implications for therapeutics and diagnostics. Prog. Neurobiol..

[250] Spinello A, Bonsignore R, Barone G, Keppler BK, Terenzi A (2016). Metal ions and metal complexes in Alzheimer’s disease. Curr. Pharm. Des..

[251] Lovell MA, Robertson JD, Teesdale WJ, Campbell JL, Markesbery WR (1998). Copper, iron and zinc in Alzheimer’s disease senile plaques. J. Neurol. Sci..

[252] Dong J (2003). Metal binding and oxidation of amyloid-beta within isolated senile plaque cores: Raman microscopic evidence. Biochemistry.

[253] Siotto M, Bucossi S, Squitti R (2010). Copper status abnormalities and how to measure them in neurodegenerative disorders. Recent Pat. CNS Drug Discov..

[254] Squitti R (2006). Excess of nonceruloplasmin serum copper in AD correlates with MMSE, CSF [beta]-amyloid, and h-tau. Neurology.

[255] Roberts BR, Ryan TM, Bush AI, Masters CL, Duce JA (2012). The role of metallobiology and amyloid-beta peptides in Alzheimer’s disease. J. Neurochem..

[256] Sparks DL, Schreurs BG (2003). Trace amounts of copper in water induce beta-amyloid plaques and learning deficits in a rabbit model of Alzheimer’s disease. Proc. Natl Acad. Sci. USA.

[257] Bayer TA (2003). Dietary Cu stabilizes brain superoxide dismutase 1 activity and reduces amyloid Abeta production in APP23 transgenic mice. Proc. Natl Acad. Sci. USA.

[258] Hua H (2011). Toxicity of Alzheimer’s disease-associated Abeta peptide is ameliorated in a Drosophila model by tight control of zinc and copper availability. Biol. Chem..

[259] Berg D, Youdim MB (2006). Role of iron in neurodegenerative disorders. Top. Magn. Reson. Imaging.

[260] Rodrigue KM, Haacke EM, Raz N (2011). Differential effects of age and history of hypertension on regional brain volumes and iron. NeuroImage.

[261] Callaghan MF (2014). Widespread age-related differences in the human brain microstructure revealed by quantitative magnetic resonance imaging. Neurobiol. Aging.

[262] Ward RJ, Zucca FA, Duyn JH, Crichton RR, Zecca L (2014). The role of iron in brain ageing and neurodegenerative disorders. Lancet Neurol..

[263] Hare DJ (2015). Is early-life iron exposure critical in neurodegeneration?. Nat. Rev..

[264] Goodman L (1953). Alzheimer’s disease; a clinico-pathologic analysis of twenty-three cases with a theory on pathogenesis. J. Nerv. Ment. Dis..

[265] Bartzokis G (1994). In vivo evaluation of brain iron in Alzheimer’s disease and normal subjects using MRI. Biol. Psychiatry.

[266] Bartzokis G, Tishler TA (2000). MRI evaluation of basal ganglia ferritin iron and neurotoxicity in Alzheimer’s and Huntingon's disease. Cell. Mol. Biol..

[267] Ding B (2009). Correlation of iron in the hippocampus with MMSE in patients with Alzheimer’s disease. J. Magn. Reson. Imaging.

[268] Pfefferbaum A, Adalsteinsson E, Rohlfing T, Sullivan EV (2009). MRI estimates of brain iron concentration in normal aging: comparison of field-dependent (FDRI) and phase (SWI) methods. NeuroImage.

[269] Luo Z (2013). The correlation of hippocampal T2-mapping with neuropsychology test in patients with Alzheimer’s disease. PloS ONE.

[270] Ghadery C (2015). R2* mapping for brain iron: associations with cognition in normal aging. Neurobiol. Aging.

[271] Langkammer C, Ropele S, Pirpamer L, Fazekas F, Schmidt R (2014). MRI for iron mapping in Alzheimer’s disease. Neurodegener Dis..

[272] Tao Y, Wang Y, Rogers JT, Wang F (2014). Perturbed iron distribution in Alzheimer’s disease serum, cerebrospinal fluid, and selected brain regions: a systematic review and meta-analysis. J. Alzheimer’s. Dis..

[273] Belaidi AA, Bush AI (2016). Iron neurochemistry in Alzheimer’s disease and Parkinson's disease: targets for therapeutics. J. Neurochem..

[274] Lane DJR, Ayton S, Bush AI (2018). Iron and Alzheimer’s disease: an update on emerging mechanisms. J. Alzheimer’s. Dis..

[275] Ke Y, Ming Qian Z (2003). Iron misregulation in the brain: a primary cause of neurodegenerative disorders. Lancet Neurol..

[276] Qian ZM, Shen X (2001). Brain iron transport and neurodegeneration. Trends Mol. Med..

[277] Qian ZM, Wang Q (1998). Expression of iron transport proteins and excessive iron accumulation in the brain in neurodegenerative disorders. Brain Res..

[278] Becerril-Ortega J, Bordji K, Freret T, Rush T, Buisson A (2014). Iron overload accelerates neuronal amyloid-beta production and cognitive impairment in transgenic mice model of Alzheimer’s disease. Neurobiol. Aging.

[279] Rogers JT (2008). Iron and the translation of the amyloid precursor protein (APP) and ferritin mRNAs: riboregulation against neural oxidative damage in Alzheimer’s disease. Biochemical Soc. Trans..

[280] Smith MA, Harris PL, Sayre LM, Perry G (1997). Iron accumulation in Alzheimer disease is a source of redox-generated free radicals. Proc. Natl Acad. Sci. USA.

[281] Faux NG (2014). An anemia of Alzheimer’s disease. Mol. Psychiatry.

[282] Atkinson A, Winge DR (2009). Metal acquisition and availability in the mitochondria. Chem. Rev..

[283] Chung SD, Sheu JJ, Kao LT, Lin HC, Kang JH (2014). Dementia is associated with iron-deficiency anemia in females: a population-based study. J. Neurol. Sci..

[284] Dixit R, Ross JL, Goldman YE, Holzbaur EL (2008). Differential regulation of dynein and kinesin motor proteins by tau. Science.

[285] Binder LI, Frankfurter A, Rebhun LI (1985). The distribution of tau in the mammalian central nervous system. J. Cell Biol..

[286] Kempf M, Clement A, Faissner A, Lee G, Brandt R (1996). Tau binds to the distal axon early in development of polarity in a microtubule- and microfilament-dependent manner. J. Neurosci..

[287] Black MM, Slaughter T, Moshiach S, Obrocka M, Fischer I (1996). Tau is enriched on dynamic microtubules in the distal region of growing axons. J. Neurosci..

[288] Liu JL, Fan YG, Yang ZS, Wang ZY, Guo C (2018). Iron and Alzheimer’s disease: from pathogenesis to therapeutic implications. Front. Neurosci..

[289] Exley C (2005). The aluminium-amyloid cascade hypothesis and Alzheimer’s disease. Sub-Cell. Biochem..

[290] Zatta P, Drago D, Bolognin S, Sensi SL (2009). Alzheimer’s disease, metal ions and metal homeostatic therapy. Trends Pharmacol. Sci..

[291] Rogers JT (1999). Translation of the alzheimer amyloid precursor protein mRNA is up-regulated by interleukin-1 through 5'-untranslated region sequences. J. Biol. Chem..

[292] Rogers JT (2002). An iron-responsive element type II in the 5'-untranslated region of the Alzheimer’s amyloid precursor protein transcript. J. Biol. Chem..

[293] Tammela T, Petrova TV, Alitalo K (2005). Molecular lymphangiogenesis: new players. Trends Cell Biol..

[294] Alitalo K, Tammela T, Petrova TV (2005). Lymphangiogenesis in development and human disease. Nature.

[295] Aspelund A (2015). A dural lymphatic vascular system that drains brain interstitial fluid and macromolecules. J. Exp. Med..

[296] Louveau A (2015). Structural and functional features of central nervous system lymphatic vessels. Nature.

[297] Absinta, M. et al. Human and nonhuman primate meninges harbor lymphatic vessels that can be visualized noninvasively by MRI. *eLife* **6.**10.7554/eLife.29738 (2017). 10.7554/eLife.29738 PMC5626482 28971799

[298] Sweeney MD, Sagare AP, Zlokovic BV (2018). Blood-brain barrier breakdown in Alzheimer disease and other neurodegenerative disorders. Nat. Rev. Neurol..

[299] Zhao Z (2015). Central role for PICALM in amyloid-beta blood-brain barrier transcytosis and clearance. Nat. Neurosci..

[300] Yang L (2013). Evaluating glymphatic pathway function utilizing clinically relevant intrathecal infusion of CSF tracer. J. Transl. Med..

[301] Thrane AS, Rangroo Thrane V, Nedergaard M (2014). Drowning stars: reassessing the role of astrocytes in brain edema. Trends Neurosci..

[302] Iliff JJ, Nedergaard M (2013). Is there a cerebral lymphatic system?. Stroke.

[303] Jessen NA, Munk AS, Lundgaard I, Nedergaard M (2015). The glymphatic system: a beginner's guide. Neurochem. Res..

[304] Shibata M (2000). Clearance of Alzheimer’s amyloid-ss(1-40) peptide from brain by LDL receptor-related protein-1 at the blood-brain barrier. J. Clin. Investig..

[305] Iliff JJ (2012). A paravascular pathway facilitates CSF flow through the brain parenchyma and the clearance of interstitial solutes, including amyloid beta. Sci. Transl. Med..

[306] Mestre, H. et al. Aquaporin-4-dependent glymphatic solute transport in the rodent brain. *eLife* **7**, 10.7554/eLife.40070 (2018). 10.7554/eLife.40070 PMC6307855 30561329

[307] Da Mesquita S, Fu Z, Kipnis J (2018). The meningeal lymphatic system: a new player in neurophysiology. Neuron.

[308] Da Mesquita S (2018). Functional aspects of meningeal lymphatics in ageing and Alzheimer’s disease. Nature.

[309] Sjogren T, Sjogren H, Lindgren AG (1952). Morbus Alzheimer and morbus pick; a genetic, clinical and patho-anatomical study. Acta Psychiatr. Neurol. Scand. Suppl..

[310] Middleton PJ, Petric M, Kozak M, Rewcastle NB, McLachlan DR (1980). Herpes-simplex viral genome and senile and presenile dementias of Alzheimer and pick. Lancet.

[311] McNamara J, Murray TA (2016). Connections between herpes simplex virus type 1 and Alzheimer’s disease pathogenesis. Curr. Alzheimer Res..

[312] Itzhaki RF (2014). Herpes simplex virus type 1 and Alzheimer’s disease: increasing evidence for a major role of the virus. Front. Aging Neurosci..

[313] Itzhaki RF (2016). Herpes and Alzheimer’s disease: subversion in the central nervous system and how it might be halted. J. Alzheimer’s. Dis..

[314] Carbone I (2014). Herpes virus in Alzheimer’s disease: relation to progression of the disease. Neurobiol. Aging.

[315] Itzhaki RF (2016). Microbes and Alzheimer’s disease. J. Alzheimer’s. Dis..

[316] Mastroeni D (2018). Laser-captured microglia in the Alzheimer’s and Parkinson's brain reveal unique regional expression profiles and suggest a potential role for hepatitis B in the Alzheimer’s brain. Neurobiol. Aging.

[317] Itzhaki RF (2017). Herpes simplex virus type 1 and Alzheimer’s disease: possible mechanisms and signposts. FASEB J..

[318] Lovheim H, Gilthorpe J, Adolfsson R, Nilsson LG, Elgh F (2015). Reactivated herpes simplex infection increases the risk of Alzheimer’s disease.. Alzheimers Dement..

[319] Lovheim H (2015). Herpes simplex infection and the risk of Alzheimer’s disease: a nested case-control study. Alzheimer’s. Dement..

[320] Westman G (2017). Decreased HHV-6 IgG in Alzheimer’s disease. Front. Neurol..

[321] Readhead, B. et al. Multiscale analysis of independent Alzheimer’s cohorts finds disruption of molecular, genetic, and clinical networks by human herpesvirus. *Neuron*. **99**, 64–82 (2018). 10.1016/j.neuron.2018.05.023 PMC6551233 29937276

[322] Kumar DK (2016). Amyloid-beta peptide protects against microbial infection in mouse and worm models of Alzheimer’s disease. Sci. Transl. Med..

[323] Soscia SJ (2010). The Alzheimer’s disease-associated amyloid beta-protein is an antimicrobial peptide. PloS ONE.

[324] Mohr AM, Mott JL (2015). Overview of microRNA biology. Semin. Liver Dis..

[325] Woldemichael BT, Mansuy IM (2016). Micro-RNAs in cognition and cognitive disorders: potential for novel biomarkers and therapeutics. Biochemical Pharmacol..

[326] Harden JT, Krams SM (2018). Micro-RNAs in transplant tolerance. Curr. Opin. Organ Transpl..

[327] Ul Hussain M (2012). Micro-RNAs (miRNAs): genomic organisation, biogenesis and mode of action. Cell Tissue Res..

[328] Wang WX (2008). The expression of microRNA miR-107 decreases early in Alzheimer’s disease and may accelerate disease progression through regulation of beta-site amyloid precursor protein-cleaving enzyme 1. J. Neurosci..

[329] Hebert SS (2008). Loss of microRNA cluster miR-29a/b-1 in sporadic Alzheimer’s disease correlates with increased BACE1/beta-secretase expression. Proc. Natl Acad. Sci. USA.

[330] Liu B (2006). Preparation and identification of a series of mannose glucuronic acid oligosaccharides. Chem. J. Chin. Univ..

[331] Gao Y, Zhang L, Jiao W (2019). Marine glycan-derived therapeutics in China. Prog. Mol. Biol. Transl. Sci..

[332] Kong LN (2005). Effects of acidic oligose on differentially expressed genes in the mice model of Alzheimer’s disease by microarray. Acta Pharm. Sin..

[333] M G (2017). GV-971, a new drug against Alzheimer’s disease. Chin. J. Pharmacol Toxicol..

[334] Mortimer JA (2012). Changes in brain volume and cognition in a randomized trial of exercise and social interaction in a community-based sample of non-demented Chinese elders. J. Alzheimer’s. Dis..

[335] Fotenos AF, Snyder AZ, Girton LE, Morris JC, Buckner RL (2005). Normative estimates of cross-sectional and longitudinal brain volume decline in aging and AD. Neurology.

[336] Freeman SH (2008). Preservation of neuronal number despite age-related cortical brain atrophy in elderly subjects without Alzheimer disease. J. Neuropathol. Exp. Neurol..

[337] Meyer D, Bonhoeffer T, Scheuss V (2014). Balance and stability of synaptic structures during synaptic plasticity. Neuron.

[338] Spruston N (2008). Pyramidal neurons: dendritic structure and synaptic integration. Nat. Rev. Neurosci..

[339] Hart MP, Hobert O (2018). Neurexin controls plasticity of a mature, sexually dimorphic neuron. Nature.

[340] Sala-Llonch R (2017). Inflammation, amyloid, and atrophy in the aging brain: relationships with longitudinal changes in cognition. J. Alzheimer’s. Dis..

[341] Paz Soldan MM (2015). Correlation of brain atrophy, disability, and spinal cord atrophy in a murine model of multiple sclerosis. J. Neuroimaging.

[342] Last N, Tufts E, Auger LE (2017). The effects of meditation on grey matter atrophy and neurodegeneration: a systematic review. J. Alzheimer’s. Dis..

[343] Moran C (2013). Brain atrophy in type 2 diabetes: regional distribution and influence on cognition. Diabetes Care.

[344] Chapleau M, Aldebert J, Montembeault M, Brambati SM (2016). Atrophy in Alzheimer’s disease and semantic dementia: an ALE meta-analysis of voxel-based morphometry studies. J. Alzheimer’s. Dis..

[345] Pini L (2016). Brain atrophy in Alzheimer’s disease and aging. Ageing Res. Rev..

[346] Risacher SL (2017). Alzheimer disease brain atrophy subtypes are associated with cognition and rate of decline. Neurology.

[347] Allemang-Grand R (2015). Altered brain development in an early-onset murine model of Alzheimer’s disease. Neurobiol. Aging.

[348] Jack CR (1998). Rate of medial temporal lobe atrophy in typical aging and Alzheimer’s disease. Neurology.

[349] Ingvar DH, Risberg J, Schwartz MS (1975). Evidence of subnormal function of association cortex in presenile dementia. Neurology.

[350] Ferris SH (1980). Positron emission tomography in the study of aging and senile dementia. Neurobiol. Aging.

[351] Hirono N, Kitagaki H, Kazui H, Hashimoto M, Mori E (2000). Impact of white matter changes on clinical manifestation of Alzheimer’s disease: a quantitative study. Stroke.

[352] Liu ZD, Zhang S, Hao JJ, Xie TR, Kang JS (2016). Cellular model of neuronal atrophy induced by DYNC1I1 deficiency reveals protective roles of RAS-RAF-MEK signaling. Protein Cell.

[353] Mizushima N, Levine B, Cuervo AM, Klionsky DJ (2008). Autophagy fights disease through cellular self-digestion. Nature.

[354] Hayashi-Nishino M (2009). A subdomain of the endoplasmic reticulum forms a cradle for autophagosome formation. Nat. Cell Biol..

[355] Klionsky DJ (2007). Autophagy: from phenomenology to molecular understanding in less than a decade. Nat. Rev. Mol. Cell Biol..

[356] Wirawan E, Vanden Berghe T, Lippens S, Agostinis P, Vandenabeele P (2012). Autophagy: for better or for worse. Cell Res..

[357] Mizushima N, Komatsu M (2011). Autophagy: renovation of cells and tissues. Cell.

[358] Wong E, Cuervo AM (2010). Autophagy gone awry in neurodegenerative diseases. Nat. Neurosci..

[359] Nixon RA, Yang DS, Lee JH (2008). Neurodegenerative lysosomal disorders: a continuum from development to late age. Autophagy.

[360] Winslow AR, Rubinsztein DC (2008). Autophagy in neurodegeneration and development. Biochimica et. Biophysica Acta.

[361] Harris H, Rubinsztein DC (2011). Control of autophagy as a therapy for neurodegenerative disease. Nat. Rev. Neurol..

[362] Nixon RA (2013). The role of autophagy in neurodegenerative disease. Nat. Med..

[363] Komatsu M (2006). Loss of autophagy in the central nervous system causes neurodegeneration in mice. Nature.

[364] Boland B (2008). Autophagy induction and autophagosome clearance in neurons: relationship to autophagic pathology in Alzheimer’s disease. J. Neurosci..

[365] Lee S, Sato Y, Nixon RA (2011). Lysosomal proteolysis inhibition selectively disrupts axonal transport of degradative organelles and causes an Alzheimer’s-like axonal dystrophy. J. Neurosci..

[366] Salminen A (2013). Impaired autophagy and APP processing in Alzheimer’s disease: the potential role of Beclin 1 interactome. Prog. Neurobiol..

[367] Wang L (2017). The cytoplasmic nuclear shuttling of Beclin 1 in neurons with Alzheimer’s disease-like injury. Neurosci. Lett..

[368] Pickford F (2008). The autophagy-related protein beclin 1 shows reduced expression in early Alzheimer disease and regulates amyloid beta accumulation in mice. J. Clin. Investig..

[369] Xiao FH (2018). Transcriptome evidence reveals enhanced autophagy-lysosomal function in centenarians. Genome Res..

[370] Luo, R. et al. Activation of PPARA-mediated autophagy reduces Alzheimer disease-like pathology and cognitive decline in a murine model. *Autophagy*, 1–18. 10.1080/15548627.2019.1596488 (2019). 10.1080/15548627.2019.1596488 PMC6984507 30898012

[371] Kim SK (2006). ERK1/2 is an endogenous negative regulator of the gamma-secretase activity. FASEB J..

[372] Cirrito JR (2011). Serotonin signaling is associated with lower amyloid-beta levels and plaques in transgenic mice and humans. Proc. Natl Acad. Sci. USA.

[373] Origlia N, Arancio O, Domenici L, Yan SS (2009). MAPK, beta-amyloid and synaptic dysfunction: the role of RAGE. Expert Rev. Neurotherapeutics.

[374] Nicotra A (2005). MAPKs mediate the activation of cytosolic phospholipase A2 by amyloid beta(25-35) peptide in bovine retina pericytes. Biochimica et. Biophysica Acta.

[375] Zempel H, Thies E, Mandelkow E, Mandelkow EM (2010). Abeta oligomers cause localized Ca(2+) elevation, missorting of endogenous Tau into dendrites, Tau phosphorylation, and destruction of microtubules and spines. J. Neurosci..

[376] Acosta-Cabronero J (2011). Atrophy, hypometabolism and white matter abnormalities in semantic dementia tell a coherent story. Brain.

[377] La Joie R (2012). Region-specific hierarchy between atrophy, hypometabolism, and beta-amyloid (Abeta) load in Alzheimer’s disease dementia. J. Neurosci..

[378] Costantini LC, Barr LJ, Vogel JL, Henderson ST (2008). Hypometabolism as a therapeutic target in Alzheimer’s disease. BMC Neurosci..

[379] Erecinska M, Silver IA (1989). ATP and brain function. J. Cereb. Blood Flow. Metab..

[380] Arnold SE (2018). Brain insulin resistance in type 2 diabetes and Alzheimer disease: concepts and conundrums. *Nature reviews*. Neurology.

[381] Cunha RA, Ribeiro JA (2000). ATP as a presynaptic modulator. Life Sci..

[382] Cisneros-Mejorado A, Perez-Samartin A, Gottlieb M, Matute C (2015). ATP signaling in brain: release, excitotoxicity and potential therapeutic targets. Cell. Mol. Neurobiol..

[383] Weise CM (2018). Left lateralized cerebral glucose metabolism declines in amyloid-beta positive persons with mild cognitive impairment. NeuroImage. Clin..

[384] Croteau E (2018). A cross-sectional comparison of brain glucose and ketone metabolism in cognitively healthy older adults, mild cognitive impairment and early Alzheimer’s disease. Exp. Gerontol..

[385] Neth BJ, Craft S (2017). Insulin resistance and Alzheimer’s disease: bioenergetic linkages. Front. Aging Neurosci..

[386] de Leon MJ (1983). Positron emission tomographic studies of aging and Alzheimer disease. AJNR Am. J. Neuroradiol..

[387] Di Domenico F, Barone E, Perluigi M, Butterfield DA (2017). The triangle of death in Alzheimer’s disease brain: the aberrant cross-talk among energy metabolism, mammalian target of rapamycin signaling, and protein homeostasis revealed by redox proteomics. Antioxid. Redox Signal..

[388] Szablewski L (2017). Glucose transporters in brain: in health and in Alzheimer’s disease. J. Alzheimer’s. Dis.

[389] Green DR, Kroemer G (2004). The pathophysiology of mitochondrial cell death. Science.

[390] Danial NN, Korsmeyer SJ (2004). Cell death: critical control points. Cell.

[391] Wallace DC, Fan W, Procaccio V (2010). Mitochondrial energetics and therapeutics. Annu. Rev. Pathol..

[392] Mishra P, Chan DC (2016). Metabolic regulation of mitochondrial dynamics. J. Cell Biol..

[393] Kang JS (2008). Docking of axonal mitochondria by syntaphilin controls their mobility and affects short-term facilitation. Cell.

[394] Wallace DC (2005). The mitochondrial genome in human adaptive radiation and disease: on the road to therapeutics and performance enhancement. Gene.

[395] van der Bliek AM, Sedensky MM, Morgan PG (2017). Cell biology of the mitochondrion. Genetics.

[396] Oyewole AO, Birch-Machin MA (2015). Mitochondria-targeted antioxidants. FASEB J..

[397] Grimm A, Mensah-Nyagan AG, Eckert A (2016). Alzheimer, mitochondria and gender. Neurosci. Biobehav. Rev..

[398] Swerdlow RH (2017). Mitochondria, cybrids, aging, and Alzheimer’s disease. Prog. Mol. Biol. Transl. Sci..

[399] Cardoso S, Seica RM, Moreira PI (2017). Mitochondria as a target for neuroprotection: implications for Alzheimer s disease. Expert Rev. Neurotherapeutics.

[400] Chetelat G (2016). Atrophy, hypometabolism and clinical trajectories in patients with amyloid-negative Alzheimer’s disease. Brain.

[401] White E, Mehnert JM, Chan CS (2015). Autophagy, metabolism, and cancer. Clin. Cancer Res..

[402] Perez-Caballero L, Torres-Sanchez S, Bravo L, Mico JA, Berrocoso E (2014). Fluoxetine: a case history of its discovery and preclinical development. Expert Opin. Drug Discov..

[403] Meltzer CC (1998). Serotonin in aging, late-life depression, and Alzheimer’s disease: the emerging role of functional imaging. Neuropsychopharmacology.

[404] Hajszan T, MacLusky NJ, Leranth C (2005). Short-term treatment with the antidepressant fluoxetine triggers pyramidal dendritic spine synapse formation in rat hippocampus. Eur. J. Neurosci..

[405] Vakili K (2000). Hippocampal volume in primary unipolar major depression: a magnetic resonance imaging study. Biol. Psychiatry.

[406] Santarelli L (2003). Requirement of hippocampal neurogenesis for the behavioral effects of antidepressants. Science.

[407] Chen S, Owens GC, Crossin KL, Edelman DB (2007). Serotonin stimulates mitochondrial transport in hippocampal neurons. Mol. Cell. Neurosci..

[408] Mendez-David I (2014). Rapid anxiolytic effects of a 5-HT(4) receptor agonist are mediated by a neurogenesis-independent mechanism. Neuropsychopharmacology.

[409] Imoto Y (2015). Role of the 5-HT4 receptor in chronic fluoxetine treatment-induced neurogenic activity and granule cell dematuration in the dentate gyrus. Mol. Brain.

[410] Reynolds GP (1995). 5-Hydroxytryptamine (5-HT)4 receptors in post mortem human brain tissue: distribution, pharmacology and effects of neurodegenerative diseases. Br. J. Pharmacol..

[411] Sachs B. D., Caron M. G. (2014). Chronic Fluoxetine Increases Extra-Hippocampal Neurogenesis in Adult Mice. International Journal of Neuropsychopharmacology.

[412] Bath KG (2012). BDNF Val66Met impairs fluoxetine-induced enhancement of adult hippocampus plasticity. Neuropsychopharmacology.

[413] Jin HJ (2017). Alleviative effects of fluoxetine on depressive-like behaviors by epigenetic regulation of BDNF gene transcription in mouse model of post-stroke depression. Sci. Rep..

[414] Nigam SM (2017). Exercise and BDNF reduce Abeta production by enhancing alpha-secretase processing of APP. J. Neurochem..

[415] Farrelly LA (2019). Histone serotonylation is a permissive modification that enhances TFIID binding to H3K4me3. Nature.

[416] Szasz BK (2007). Direct inhibitory effect of fluoxetine on N-methyl-D-aspartate receptors in the central nervous system. Biol. Psychiatry.

[417] Jin L (2017). Long-term ameliorative effects of the antidepressant fluoxetine exposure on cognitive deficits in 3 x TgAD mice. Mol. Neurobiol..

[418] Hashimoto K (2015). Activation of sigma-1 receptor chaperone in the treatment of neuropsychiatric diseases and its clinical implication. J. Pharmacol. Sci..

[419] Matsuno K, Matsunaga K, Senda T, Mita S (1993). Increase in extracellular acetylcholine level by sigma ligands in rat frontal cortex. J. Pharmacol. Exp. Ther..

[420] Hashimoto K (2009). Sigma-1 receptors and selective serotonin reuptake inhibitors: clinical implications of their relationship. Cent. Nerv. Syst. Agents Med. Chem..

[421] Hayashi T, Su TP (2007). Sigma-1 receptor chaperones at the ER-mitochondrion interface regulate Ca(2+) signaling and cell survival. Cell.

[422] Tarasov AI, Griffiths EJ, Rutter GA (2012). Regulation of ATP production by mitochondrial Ca^2+^. Cell Calcium.

[423] Xie TR, Liu CF, Kang JS (2017). Sympathetic transmitters control thermogenic efficacy of brown adipocytes by modulating mitochondrial complex V. Signal Transduct. Target. Ther..

[424] Xie, Y., Liu, P.-P., Lian, Y.-J., Liu, H.-b., Kang, J.-S. The effect of selective serotonin reuptake inhibitors on cognitive function in patients with Alzheimer’s disease and vascular dementia: focusing on fluoxetine with long follow-up periods. *Signal Transduct. Targeted Ther.*10.1038/s41392-019-0064-7 (2019). 10.1038/s41392-019-0064-7 PMC6799811 31637010

[425] Matrisciano F (2009). Changes in BDNF serum levels in patients with major depression disorder (MDD) after 6 months treatment with sertraline, escitalopram, or venlafaxine. J. Psychiatr. Res..

[426] Aboukhatwa M, Dosanjh L, Luo Y (2010). Antidepressants are a rational complementary therapy for the treatment of Alzheimer’s disease. Mol. Neurodegener..

[427] Kaether C, Haass C, Steiner H (2006). Assembly, trafficking and function of gamma-secretase. Neurodegener. Dis..

[428] Lu P (2014). Three-dimensional structure of human gamma-secretase. Nature.

[429] Sun L, Zhou R, Yang G, Shi Y (2017). Analysis of 138 pathogenic mutations in presenilin-1 on the in vitro production of Abeta42 and Abeta40 peptides by gamma-secretase. Proc. Natl Acad. Sci. USA.

[430] Kelleher RJ, Shen J (2017). Presenilin-1 mutations and Alzheimer’s disease. Proc. Natl Acad. Sci. USA.

[431] Saura CA (2004). Loss of presenilin function causes impairments of memory and synaptic plasticity followed by age-dependent neurodegeneration. Neuron.

[432] De Strooper B (1999). A presenilin-1-dependent gamma-secretase-like protease mediates release of Notch intracellular domain. Nature.

[433] Zheng J (2012). Conditional deletion of Notch1 and Notch2 genes in excitatory neurons of postnatal forebrain does not cause neurodegeneration or reduction of Notch mRNAs and proteins. J. Biol. Chem..

[434] Ni CY, Murphy MP, Golde TE, Carpenter G (2001). gamma-Secretase cleavage and nuclear localization of ErbB-4 receptor tyrosine kinase. Science.

[435] Marambaud P (2002). A presenilin-1/gamma-secretase cleavage releases the E-cadherin intracellular domain and regulates disassembly of adherens junctions. EMBO J..

[436] Marambaud P (2003). A CBP binding transcriptional repressor produced by the PS1/epsilon-cleavage of N-cadherin is inhibited by PS1 FAD mutations. Cell.

[437] Georgakopoulos A (2006). Metalloproteinase/Presenilin1 processing of ephrinB regulates EphB-induced Src phosphorylation and signaling. EMBO J..

[438] Lammich S (2002). Presenilin-dependent intramembrane proteolysis of CD44 leads to the liberation of its intracellular domain and the secretion of an Abeta-like peptide. J. Biol. Chem..

[439] May P, Reddy YK, Herz J (2002). Proteolytic processing of low density lipoprotein receptor-related protein mediates regulated release of its intracellular domain. J. Biol. Chem..

[440] Goodnick PJ, Chaudry T, Artadi J, Arcey S (2000). Women's issues in mood disorders. Expert Opin. Pharmacother..

[441] Mazure CM, Swendsen J (2016). Sex differences in Alzheimer’s disease and other dementias. Lancet Neurol..

[442] Austad SN, Fischer KE (2016). Sex differences in lifespan. Cell Metab..

[443] Brooks-Wilson AR (2013). Genetics of healthy aging and longevity. Hum. Genet..

[444] Breitner JC (1999). APOE-epsilon4 count predicts age when prevalence of AD increases, then declines: the Cache County Study. Neurology.

[445] Park S, Choi SG, Yoo SM, Son JH, Jung YK (2014). Choline dehydrogenase interacts with SQSTM1/p62 to recruit LC3 and stimulate mitophagy. Autophagy.

[446] Zhang Y (2019). Listeria hijacks host mitophagy through a novel mitophagy receptor to evade killing. Nat. Immunol..

[447] Chen Z, Siraj S, Liu L, Chen Q (2017). MARCH5-FUNDC1 axis fine-tunes hypoxia-induced mitophagy. Autophagy.

[448] Princely Abudu Y (2019). NIPSNAP1 and NIPSNAP2 act as “Eat Me” signals for mitophagy. Dev. Cell.

[449] Ha J, Guan KL, Kim J (2015). AMPK and autophagy in glucose/glycogen metabolism. Mol. Asp. Med..

[450] Wan W (2017). mTORC1 Phosphorylates Acetyltransferase p300 to Regulate Autophagy and Lipogenesis. Mol. Cell.

[451] Zheng M (2011). Inactivation of Rheb by PRAK-mediated phosphorylation is essential for energy-depletion-induced suppression of mTORC1. Nat. Cell Biol..

[452] Levin-Salomon V, Bialik S, Kimchi A (2014). DAP-kinase and autophagy. Apoptosis.

[453] Torres-Quiroz F, Filteau M, Landry CR (2015). Feedback regulation between autophagy and PKA. Autophagy.

[454] Su H (2017). VPS34 acetylation controls its lipid kinase activity and the initiation of canonical and non-canonical autophagy. Mol. Cell.

[455] Stadtman ER (1993). Oxidation of free amino acids and amino acid residues in proteins by radiolysis and by metal-catalyzed reactions. Annu. Rev. Biochem..

[456] Xu W, Barrientos T, Andrews NC (2013). Iron and copper in mitochondrial diseases. Cell Metab..

[457] Delnomdedieu M (2016). First-in-human safety and long-term exposure data for AAB-003 (PF-05236812) and biomarkers after intravenous infusions of escalating doses in patients with mild to moderate Alzheimer’s disease. Alzheimer’s. Res. Ther..

