# Author Correction: History and progress of hypotheses and clinical trials for Alzheimer’s disease

**DOI:** 10.1038/s41392-019-0071-8

**Published:** 2019-09-23

**Authors:** Pei-Pei Liu, Yi Xie, Xiao-Yan Meng, Jian-Sheng Kang

**Affiliations:** Clinical Systems Biology Laboratories, The First Affiliated Hospital of Zhengzhou University, Zhengzhou, 450052 Henan China

**Keywords:** Neuroscience, Neurological disorders

**Correction to:** *Signal Transduction and Targeted Therapy* 10.1038/s41392-019-0063-8, published online 23 August 2019

Since the publication of this Review Article, we noticed several mistakes in Table 2 and the Perspective section that need to be corrected immediately.

**Table 2:** Current status of selected AD drugs in clinical trials

Drug	Developer	Mechanism of action	Stage	NCT number (https://clinicaltrials.gov)
AAB-003 (PF‑05236812)	Janssen/Pfizer	Aβ-specific mAb	Phase I (completed)^457^	NCT01193608
GV-971	Shanghai Green Valley Pharmaceutical Co., Ltd.	Mannose oligosaccharide diacid	Phase III (completed in China)	NCT02293915

In Table 2, the status of AAB-003 Phase I trial is completed. In addition, the status of GV-971 Phase III trial is completed in China. The correct portion of Table 2 is displayed as below.

In the perspective section, the sigma-1 receptor activator Anavex 2-73 has entered a phase III clinical trial but it was not granted fast-track status by the FDA. Therefore, the texts should be corrected to “In addition, fluoxetine can bind to the endoplasmic reticulum protein sigma-1 receptor.^418^ Sigma-1 receptor ligands can enhance acetylcholine secretion.^419,420^ The sigma-1 receptor is located in the mitochondrion-associated ER membrane so that the activation of the sigma-1 receptor can prolong Ca^2+^ signaling in mitochondria.^421^ Consequently, the local and specific elevation of [Ca^2+^] in the mitochondrial matrix can enhance ATP synthesis,^422,423^ which ameliorates hypometabolism. Interestingly, Anavex 2-73, a sigma-1 receptor activator is now in phase III clinical trial.”

The key messages of the article are not affected by these corrections. We apologize for these inadvertent mistakes.

